# Maternal diet deficient in riboflavin induces embryonic death associated with alterations in the hepatic proteome of duck embryos

**DOI:** 10.1186/s12986-019-0345-8

**Published:** 2019-03-14

**Authors:** Jing Tang, Jian Hu, Ming Xue, Zhanbao Guo, Ming Xie, Bo Zhang, Zhengkui Zhou, Wei Huang, Shuisheng Hou

**Affiliations:** 1 State Key Laboratory of Animal Nutrition; Key Laboratory of Animal (Poultry) Genetics Breeding and Reproduction, Ministry of Agriculture and Rural Affairs, Institute of Animal Sciences, Chinese Academy of Agricultural Sciences, Beijing, 100193 China; 2 National Animal Husbandry Service, Beijing, 100125 China

**Keywords:** Maternal riboflavin deficiency, Embryonic death, Liver proteomics, Beta-oxidation, Electron transport chain

## Abstract

**Background:**

Maternal riboflavin deficiency (RD) induces embryonic death in poultry. The underlying mechanisms, however, remain to be established and an overview of molecular alterations at the protein level is still lacking. We investigated embryonic hepatic proteome changes induced by maternal RD to explain embryonic death.

**Methods:**

A total of 80 45-week-old breeding female ducks were divided into two groups of 40 birds each, and all birds were raised individually for 8 weeks. All the female ducks received either a RD or a riboflavin adequate (control, CON) diet, which supplemented the basal diet with 0 or 10 mg riboflavin /kg of diet respectively.

**Results:**

The riboflavin concentrations of maternal plasma and egg yolk, as well as egg hatchability declined markedly in the RD group compared to those in the CON group after 2 weeks, and declined further over time. The hepatic proteome of E13 viable embryos from 8-week fertile eggs showed that 223 proteins were upregulated and 366 proteins were downregulated (> 1.5-fold change) in the RD group compared to those in the CON group. Pathway analysis showed that differentially expressed proteins were mainly enriched in the fatty acid beta-oxidation, electron transport chain (ETC), and tricarboxylic acid (TCA) cycle. Specifically, all the proteins involved in the fatty acid beta-oxidation and ETC, as well as six out of seven proteins involved in the TCA cycle, were diminished in the RD group, indicating that these processes could be impaired by RD.

**Conclusion:**

Maternal RD leads to embryonic death of offspring and is associated with impaired energy generation processes, indicated by a number of downregulated proteins involved in the fatty acid beta-oxidation, ETC, and TCA cycle in the hepatic of duck embryos. These findings contribute to our understanding of the mechanisms of liver metabolic disorders due to maternal RD.

**Electronic supplementary material:**

The online version of this article (10.1186/s12986-019-0345-8) contains supplementary material, which is available to authorized users.

## Introduction

Riboflavin (vitamin B_2_) is an essential precursor of flavin mononucleotide (FMN) and flavin adenine dinucleotide (FAD). A number of flavin-dependent proteins that utilize FMN or/and FAD, so-called flavoproteins, participate in a range of redox reactions in the tricarboxylic acid (TCA) cycle, fatty acid beta-oxidation, amino acid degradation, and electron transport chain (ETC), among others [1, 2]. Due to its involvement in primary metabolic pathways, maternal riboflavin deficiency (RD) causes abnormal development of offspring. In mammals, insufficient riboflavin supplementation in rat dams during lactation markedly decreased riboflavin concentrations in the liver, carcass, and milk [3], and reduced the body weight of offspring by 20% [4]. In poultry, maternal RD in laying hens decreased the riboflavin concentrations in egg yolk and resulted in embryonic death [5, 6]. Inherited RD chicken embryos have delayed and aberrant feather development (clubbed down), fatty livers, and extensive cutaneous hemorrhaging, which suddenly die at mid-incubation [7–10]. An explanation of the sudden death in RD embryos is due to the inhibition of the various critical biological pathways, leading to energy depletion, lipid accumulation in the liver, and severe hypoglycemia [8, 9]. Of these, the beta-oxidation of lipids was severely impaired in RD embryos, as indicated by the reduction in the activity of acyl-CoA dehydrogenase, a flavin-dependent enzyme, and the accumulation of lipids and fatty acid oxidation intermediates in the embryonic liver [7, 9]. Cogburn et al. (2018) recently confirmed that RD in mid-stage embryos leads to a reduced expression of numerous genes involved in the beta-oxidation of lipids and energy depletion at the transcriptional level, including medium chain acyl-CoA dehydrogenase transcripts [8]. However, the underlying mechanisms of embryonic death caused by RD are unknown at the protein level-the functional relevant integration level. Here, we used a proteomic approach to investigate the effects of maternal RD on the hepatic protein levels of offspring.

## Materials and methods

### Animals ethics statement

This study, which complied with institutional and national guidelines for the care and use of animals, was approved by the Committee of Animal Experiments of the Institute of Animal Sciences, Chinese Academy of Agricultural Sciences. All efforts were made to minimize animal suffering.

### Animals and housing

This study was designed to investigate the effects of maternal RD on the hepatic protein levels of offspring using a proteomic approach. A total of 80 45-week-old breeding female white Pekin ducks (*Anas platyrhynchos*) were obtained from the Pekin duck breeding center (Chinese Academy of Agricultural Sciences) and randomly divided into two treatment groups with 10 replicates (4 birds per replicate) for each group. All female ducks were initially adapted for 2 weeks using a conventional corn-soybean meal diet supplemented with 10 mg riboflavin /kg of diet. After that, the birds received two experimental diets for 8 weeks that supplemented the basal diet with 0 or 10 mg riboflavin /kg of diet. The female ducks were fertilized by male Pekin ducks of the same age fed the experimental diet supplemented the basal diet with 10 mg riboflavin /kg of diet during the experimental period. The birds in the two treatments were housed in individual plastic cages in an environmentally controlled room and received ad libitum access to water and feed. Eighteen hours of light were provided daily from 04:00 to 22:00 h.

### Diet

All the female ducks were raised with common diets from hatch to 45 weeks of age, and all nutrients met the recommendations for ducks as established in the Nutrient Requirements of Meat-type Ducks of China [11]. The basal diet during the experimental period was riboflavin-deficient containing 1.48 mg free riboflavin/kg of diet (Table 1). The RD diet and CON diet were produced from the basal diet, which was supplemented with 0 or 10 mg crystalline riboflavin/kg diet (purity, 99%; Sigma Aldrich, MO, USA). Except for the riboflavin content of the basal experimental diet, all nutrients met the recommendations for laying ducks [11].

**Table 1:** Composition of riboflavin-deficient basal diet (g/kg as-fed)

Item	g/kg
Ingredient	
Corn	560.0
Soybean	238.0
Corn gluten meal	100.0
Limestone	70.0
Dicalcium phosphate	15.0
Vitamin and trace mineral premix ^a^	10.0
Sodium chloride	3.0
DL-Methionine	1.0
L-Lysine·HCl	3.0
Calculated composition	
Metabolizable energy ^b^, MJ/kg	11.48
Crude protein	192.9
Calcium	30.7
Nonphytate phosphorus	3.5
Lysine	10.4
Methionine	4.6
Methionine + cysteine	7.7
Threonine	7.6
Tryptophan	2.0
Arginine	10.1
Riboflavin ^c^, mg/kg	1.48

^a^Supplied per kilogram of total diet: Cu (CuSO_4_•5H_2_O), 10 mg; Fe (FeSO_4_•7H_2_O), 60 mg; Zn (ZnO), 60 mg; Mn (MnSO_4_•H_2_O), 80 mg; Se (NaSeO_3_), 0.3 mg; I (KI), 0.2 mg; choline chloride, 1000 mg; vitamin A (retinyl acetate), 10,000 IU; vitamin D_3_ (Cholcalciferol), 3000 IU; vitamin E (DL-α-tocopheryl acetate), 20 IU; vitamin K_3_ (menadione sodium bisulfate), 2 mg; thiamin (thiamin mononitrate), 2 mg; pyridoxine hydrochloride, 4 mg; cobalamin, 0.02 mg; calcium-D-pantothenate, 20 mg; nicotinic acid, 50 mg; folic acid, 1 mg; biotin, 0.2 mg

^b^The values are calculated according to the AME of ducks (Ministry of Agriculture of China, 2012)

^c^The value was analysed by high performance liquid chromatography

### Sampling

All female ducks were weighed at the start and end of the experiment. One duck from each replicate was randomly selected to collect whole blood from a wing vein twice weekly. Blood samples were collected into heparin sodium-anticoagulant tubes and centrifuged at 1520×g for 10 min to obtain plasma. Plasma was stored at − 20 °C until assayed for free riboflavin concentration. Meanwhile, eggs were collected and weighed every day during the experimental period, and egg production was recorded. Twelve eggs per group were selected randomly from different ducks on the last day of every two week period and were broken to collect yolk samples, which were then stored at − 20 °C until riboflavin analysis using HPLC. Sixteen eggs from each replicate (four eggs from each duck) were selected to measure fertility and hatchability in the incubator at weekly intervals from 1 to 8 weeks of the experiment.

During the incubation of 7-week eggs, all eggs from the RD group were examined for livability of the embryo at seven days and followed by every three days by means of transmitted light. During the incubation of 8-week eggs, one viable embryo at three embryonic ages (E13, E20, and E27) from each replicate was selected randomly. The embryos were sacrificed by cervical dislocation, then liver samples were collected and frozen in liquid nitrogen immediately, and subsequently stored at − 80 °C for further analyses.

### Riboflavincontent

The riboflavin concentration in feed, plasma, and egg yolk were determined by reversed-phase high performance liquid chromatography (HPLC) according to the methods described previously [12, 13]. Before HPLC analysis, feed and plasma samples were prepared according to the method described previously [14, 15], while egg yolk samples were prepared according to the method for animal tissue described previously [16]. The peak was identified and quantified by analysis of authentic standard (Sigma Aldrich).

### Liver lipids

Total lipids were extracted by homogenizing minced liver tissue samples in chloroform-methanol (2:1) as described previously [17]. The extracts were evaporated under a stream of nitrogen, weighed, and resuspended in chloroform-methanol (2:1) containing 0.01% butyrated hydroxytoluene. Measurements of total lipids in the liver were performed as described previously [18]. Aliquots were dried and resuspended in 1-butanol for analysis of triglyceride (TG) using commercial kits according to the manufacturer’s instructions (BioSino Bio-technology and Science Inc., Beijing, China).

### Liver proteomics

A total of six liver samples (three biological replicates per group) were used to conduct the isobaric tags for relative and absolute quantification (iTRAQ) assays. Each liver sample was ground in liquid nitrogen. The grinded powder was lysed in a solution containing 200 μl L3 buffer (50 mM Tris-Cl, pH 8, 8 M urea, 2 M thiourea, 2 M EDTA, 1 × protease inhibitors cocktails), 800 μl of ice-cold acetone, and 10 mM DTT. The suspensions were incubated at − 20 °C for 2 h. The precipitate pellets were obtained via centrifugation at 12,000×*g* for 20 min at 4 °C and subsequently resuspended in 800 μl of ice-cold acetone and 10 mM DTT. The suspensions were further centrifuged at 12,000×*g* for 20 min at 4 °C to collect the precipitated pellets, and then vacuum dried. The dried precipitated pellets were dissolved in 200 μl L3 buffer. Subsequently, total protein concentration was measured using the Bradford assay.

For each sample, 100 μg of protein was reduced, alkylated, and digested with trypsin according to the manufacturer’s protocol (Applied Biosystems, Framingham, MA, USA). Each digested sample was labelled with iTRAQ 8-plex reagents (AB Sciex, Foster City, USA) according to the manufacturer’s instructions. The RD samples were labelled with iTRAQ tags 113, 114, and 115, and the CON samples were labelled with tags 116, 117, and 118. Labelled samples were mixed and fractionated into 20 fractions by HPLC (DINOEX Ultimate 3000 BioRS, Thermo Fisher, Waltham, MA, USA) using a Durashell C18 column (5 μm, 100 Å, 4.6 × 250 mm). LC-electrospray ionization-MS/MS analysis was carried out with a Triple TOF 5600 plus system (AB SCIEX, Framingham, USA). The original MS/MS file data for identification and quantitation were analysed against the database *UniProt_Mallard_8839* using ProteinPilot Software version 4.0 (AB SCIEX). To minimize the false discovery rate, a threshold for protein identification was applied. Only unique peptides whose confidences are more than 95% were contained in the iTRAQ labelling quantification and used for further analysis.

For analysis of the proteomic results, the relative expressions of identified proteins were based on the ratio of the reporter ions of the peptides between the two groups (RD vs CON). A protein was considered differentially expressed when the protein had both a fold change (FC) greater than 1.5 and a *P*-value less than 0.05.

To enrich the differentially expressed proteins with respect to specific functional terms, the protein lists were analysed using ClueGo software (http://www.ici.upmc.fr/cluego/) with the Gene Ontology (GO) database (release date: February 2018). A pathway enrichment analysis of the differentially expressed proteins [19] was performed using ClueGo software and applying database from the Kyoto Encyclopedia of Genes and Genomes (KEGG) database (release date: February 2018).

### Statistical analyses

All the data were analysed using a T-test in SAS software (SAS Institute Inc., 2003). The variability in the data was expressed as the standard error of the means (SEM). Differences between means were considered statistically significant at *P* < 0.05. The relative expression of identified proteins was based on the ratio of the reporter ions of the peptides in the RD group to the CON group. Fold changes (FC) were used to determine whether identified proteins were enhanced (FC > 1.5) or diminished (FC < − 1.5) by RD. A fold change (FC) of 1.5 and *P* < 0.05 was set as the threshold to identify differentially proteins induced by RD.

## Results

### Maternal growth performance and embryonic growth

The final body weight of maternal ducks fed the RD diet was not different from the CON group (initial body weight: 3880 ± 264 g in the RD group vs 3863 ± 301 g in the CON group; final body weight: 3546 ± 265 g in the RD group vs 3604 ± 297 g in the CON group). Egg weight, egg production, and egg fertility of maternal ducks fed the RD diet were not different from those birds in the CON group from 1 to 8 weeks (*P* > 0.05, Additional files 1, 2, 3).

**Fig. 1:**
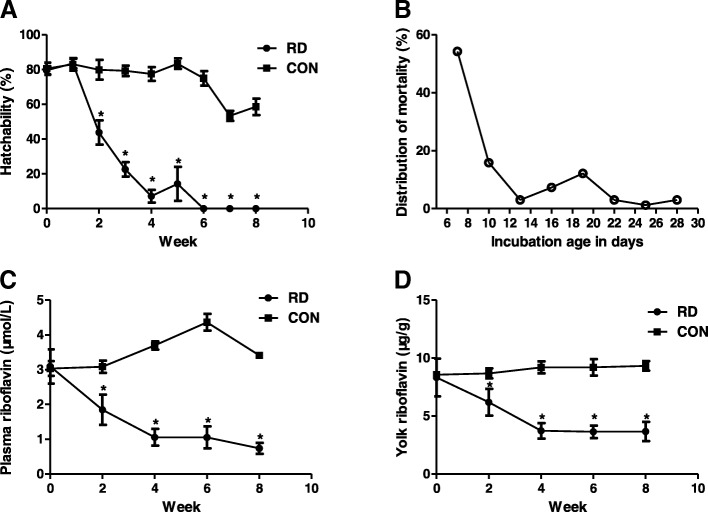
Effects of maternal riboflavin deficiency on egg hatchability, embryonic mortality, and plasma and yolk riboflavin concentration. **a** Egg hatchability in the riboflavin-deficient (RD) and the control (CON) group. **b** Time distribution curve of dead embryos from RD eggs from 7-week riboflavin-depleted ducks. A total of 164 RD eggs were included. **c** Plasma riboflavin concentration in the RD and CON maternal ducks. **d** Egg yolk riboflavin concentration in the RD and CON group. Data are means ± SEM (n = 10). An asterisk indicates a statistically significant difference between the RD and CON group at P < 0.05

The hatchability of eggs from the maternal ducks in the RD group dropped dramatically to approximately half of that in the controls after feeding the RD diet for 2 weeks (*P* < 0.05, Fig. 1a). Subsequently, from 3 to 5 weeks of the experiment, the hatchability of eggs declined further as riboflavin depletion last longer in the maternal ducks. Strikingly, the hatchability dropped to approximately zero after 6 weeks of riboflavin depletion in maternal ducks.

The embryonic mortality of all fertile RD eggs was approximately zero after 6 weeks of riboflavin depletion in maternal ducks. The curve on the time distribution of 164 dead embryos from the eggs at 7 weeks is shown in Fig. 1b. This curve displays two critical periods with the peaks on the seventh and nineteenth days of incubation, while the former was the major peak of embryonic mortality. Accumulated 54.2, 73.2, and 92.7% of RD embryos were dead within 7, 13, and 19 days of incubation, respectively, indicating that the majority of RD embryos were dead by day 13 of incubation.

### Riboflavin concentrations in maternal plasma and egg yolk

Figure 1c shows that, the RD diet reduced the riboflavin concentration in the maternal plasma by 40% of the value present in birds fed the control diet at 2 weeks of the experiment (*P* < 0.05). Afterward, maternal plasma riboflavin concentration decreased gradually to only 21.6% of the control values at 8 weeks of the experiment (*P* < 0.05). Figure 1d shows that, the riboflavin concentration in egg yolk was also decreased by RD in the maternal diet by 28.7% at the end of 2 weeks compared with the controls. Then, it was reduced further to only 42% of the control at the end of 4 weeks. Yolk riboflavin depletion was complete at 4 weeks in the RD group, stabilizing at an average of 3.7 μg riboflavin/g yolk.

### Total lipids and TG content in maternal plasma and liver, egg yolk, and embryo

Total lipids in the maternal liver, egg yolk, and embryonic liver was not affected by the riboflavin concentration in the diet (*P* > 0.05, Table 2). The TG content of the maternal liver and egg yolk from the animals fed the RD diet was not significantly different from the controls (*P* > 0.05, Table 2). However, the TG content in the liver of E27 embryos from the RD group was greater than that in the CON group (*P* < 0.05, Table 2), even though the E20 embryo was not affected (*P* > 0.05, Table 2).

**Table 2:** Total lipid and triglyceride content in tissue in the riboflavin-deficient group and the control group

Variable	RD	CON	SEM	*P*-value
Maternal liver				
Total lipid (%)	18.1	14.2	1.02	0.053
TG (mg/g)	22.5	25.2	1.07	0.230
Egg yolk				
total lipid (%)	0.28	0.28	0.006	0.592
TG (mg/g)	42.0	45.9	3.07	0.538
Embryonic liver (E20)				
Total lipid (%)	22.6	22.8	0.67	0.894
TG (mg/g)	10.6	9.24	0.55	0.226
Embryonic liver (E27)				
Total lipid (%)	17.2	15.5	0.60	0.187
TG (mg/g)	7.78^a^	4.37^b^	0.44	< 0.001

*SEM* standard error of the mean, *TG* triglyceride, *RD* riboflavin-deficient, *CON* control

^a, b^Mean values with unlike superscript letters were significantly different (*P* < 0.05). Data were analysed by a T-test. Data are expressed as the mean and pooled SEM (*n* = 10)

### Proteomic analysis of the embryonic liver

Using iTRAQ analysis, a total of 25,617 peptide spectral matches were identified, and 3801 proteins were identified in the E13 embryonic livers of two groups. Comparisons of the relative abundance of proteins from the embryonic livers of maternal ducks fed the RD diet with those fed the control diet showed that 223 proteins were enhanced and 366 diminished. The complete list of proteins regulated by maternal RD is presented in Additional file 4.

**Fig. 2:**
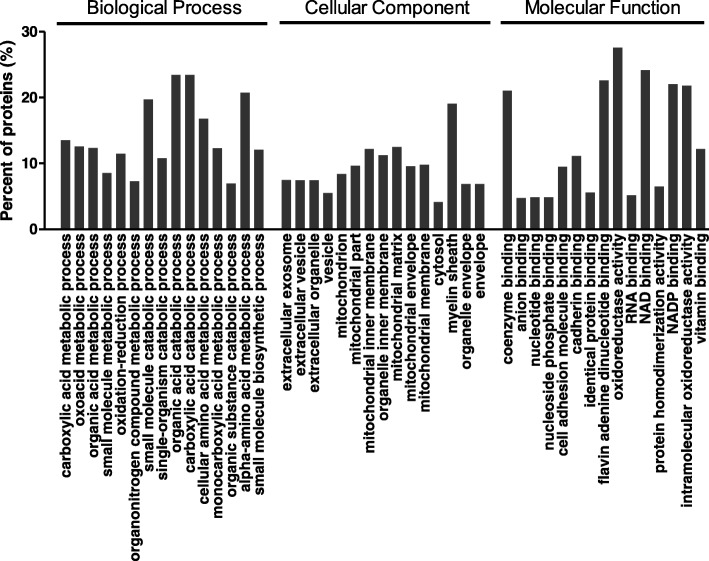
Top 15 significantly enriched biological processes, cellular components, and molecular functions

We performed GO categories of biological process, cellular component, and molecular function, and pathway analysis on the set of 589 differentially expressed proteins in livers from the RD group compared with those in livers from the CON group. As shown in Fig. 2, an enrichment analysis of the significantly enriched under the terms carboxylic acid metabolic process, oxoacid metabolic process, organic acid metabolic process, small molecule metabolic process, oxidation-reduction process, organonitrogen compound metabolic process, small molecule catabolic process, single-organism catabolic process, organic acid catabolic process, carboxylic acid catabolic process, cellular amino acid metabolic process, monocarboxylic acid metabolic process, organic substance catabolic process, alpha-amino acid metabolic process, and small molecule biosynthetic process. The top 15 enriched terms under cellular component included extracellular exosome, extracellular vesicle, extracellular organelle, vesicle, mitochondrion, mitochondrial part, mitochondrial inner membrane, organelle inner membrane, mitochondrial matrix, mitochondrial envelope, mitochondrial membrane, cytosol, myelin sheath, organelle envelope, and envelope. The top 15 enriched terms under molecular function included coenzyme binding, anion binding, nucleotide binding, nucleoside phosphate binding, cell adhesion molecule binding, cadherin binding, identical protein binding, flavin adenine dinucleotide binding, oxidoreductase activity, RNA binding, NAD binding, protein homodimerization activity, NADP binding, intramolecular oxidoreductase activity, and vitamin binding.

**Fig. 3:**
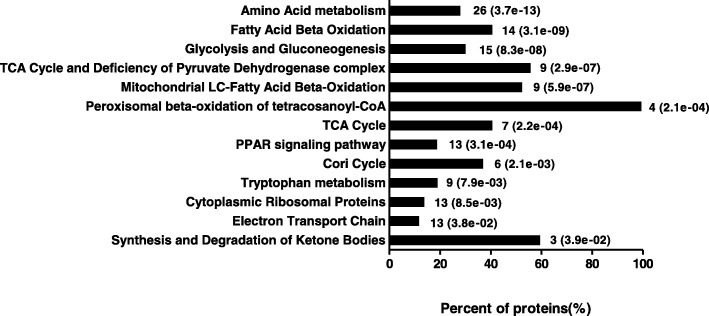
The pathway analysis by KEGG on differentially expressed proteins

The pathway analysis by KEGG on differentially expressed proteins suggested that the significantly affected pathways were amino acid metabolism, fatty acid beta-oxidation, glycolysis and gluconeogenesis, TCA cycle and deficiency of pyruvate dehydrogenase complex, mitochondrial LC-fatty acid beta-oxidation, peroxisomal beta-oxidation of tetracosanoyl-CoA, TCA cycle, PPAR signaling pathway, Cori cycle, tryptophan metabolism, cytoplasmic ribosomal proteins, electron transport chain, and synthesis and degradation of ketone bodies (Fig. 3). Since riboflavin deficiency mainly affected energy generation pathways, such as the TCA cycle, fatty acid beta-oxidation, and ETC, the proteins associated with these processes are listed in Table 3. Notably, some of these proteins are flavin-containing enzymes such as electron transfer flavoprotein subunit alpha (ETFA), flavoprotein-ubiquinone oxidoreductase (ETFDH), short-chain specific acyl-CoA dehydrogenase (ACADS), Acyl-CoA dehydrogenase long chain (ACADL), acyl-CoA dehydrogenase family member 9 (ACAD9), succinate dehydrogenase [ubiquinone] flavoprotein subunit (SDHA), dihydrolipoyl dehydrogenase (DLD), NADH dehydrogenase [ubiquinone] flavoprotein 1 (NDUFV1), NADH dehydrogenase [ubiquinone] 1 alpha subcomplex subunit 9 (NDUFA9), and NADH dehydrogenase [ubiquinone] 1 alpha subcomplex subunit 10 (NDUFA10), which were all diminished in the RD group.

**Table 3:** Differentially expressed proteins involved in the TCA cycle, fatty acid beta-oxidation, and electron transport chain

UniProtKB ID	Protein description	Protein name	% coverage	Peptides number	Unique peptides	Fold change^*^	*P*-Value	Co-factor
TCA cycle								
U3IHF6	Succinate dehydrogenase [ubiquinone] flavoprotein subunit, mitochondrial	SDHA	32.23	17	17	−5.17	2.24E-08	FAD
U3J5X3	Succinate dehydrogenase [ubiquinone] iron-sulfur subunit, mitochondrial	SDHB	41.01	11	11	−4.80	5.56E-05	
U3IR48	Dihydrolipoyl dehydrogenase	DLD	42.38	18	18	−4.54	1.91E-05	FAD
U3J6J0	Succinate--CoA ligase [ADP/GDP-forming] subunit alpha, mitochondrial	SUCLG1	36.34	7	7	−2.74	9.55E-03	
U3INY2	Dihydrolipoamide S-succinyltransferase	DLST	31.87	12	12	−1.94	2.55E-02	
R0L7Q0	Fumarate hydratase	FH	59.83	26	26	−1.62	3.80E-03	
U3I2P1	Isocitrate dehydrogenase [NAD] subunit, mitochondrial	IDH3A	28.88	8	8	3.48	6.67E-03	
Fatty acid beta-oxidation								
R0L018	Carnitine O-palmitoyltransferase I, liver isoform	CPT1A	46.10	35	35	−5.45	2.10E-14	
U3INM7	Carnitine palmitoyltransferase 2	CPT2	49.62	33	33	−5.20	5.00E-09	
U3IR48	Dihydrolipoyl dehydrogenase	DLD	42.38	18	18	−4.54	1.91E-05	FAD
R0LSV8	Electron transfer flavoprotein-ubiquinone oxidoreductase, mitochondrial	ETFDH	47.37	25	25	−4.33	4.08E-09	FAD
U3 IU30	Acyl-CoA synthetase long chain family member 1	ACSL1	58.08	43	40	−4.23	1.38E-09	
U3IAY7	Acyl-CoA dehydrogenase long chain	ACADL	50.70	26	26	−3.57	4.72E-08	FAD
U3J4Z9	Acyl-CoA synthetase long chain family member 5	ACSL5	40.85	24	21	−3.05	7.07E-07	
U3IFL0	Solute carrier family 25 member 20	SLC25A20	38.01	9	9	−2.90	4.26E-04	
U3J7F4	Electron transfer flavoprotein alpha subunit	ETFA	62.87	17	17	−2.69	1.46E-02	FAD
U3J8W0	Acyl-CoA dehydrogenase short chain	ACADS	49.30	14	14	−2.36	4.32E-03	FAD
U3IFB0	2,4-dienoyl-CoA reductase 1	DECR1	39.20	9	9	−2.32	2.32E-02	
U3I806	Trifunctional enzyme subunit alpha, mitochondrial	HADHA	53.42	39	38	−2.03	6.79E-03	
U3IK00	Carnitine palmitoyltransferase 1B	CPT1B	17.83	7	7	−1.89	2.53E-03	
U3J1J0	Acyl-CoA dehydrogenase family member 9	ACAD9	60.22	33	33	−1.73	4.14E-04	FAD
U3ILK4	Enoyl-CoA delta isomerase 1, mitochondrial	ECI1	50.00	11	11	−1.67	1.13E-02	
U3IHS8	Carnitine O-acetyltransferase	CRAT	34.71	22	22	−1.67	8.38E-04	
ETC								
U3IHF6	Succinate dehydrogenase [ubiquinone] flavoprotein subunit, mitochondrial	SDHA	32.23	17	17	−5.17	2.24E-08	FAD
U3J5X3	Succinate dehydrogenase [ubiquinone] iron-sulfur subunit, mitochondrial	SDHB	41.01	11	11	−4.80	5.56E-05	
R0K082	NADH-ubiquinone oxidoreductase 75 kDa subunit, mitochondrial	NDUFS1	55.59	34	34	−2.79	1.02E-06	
R0LIL9	ATP synthase subunit O, mitochondrial	ATP5PO	69.02	13	13	−2.56	4.03E-03	
U3IKH0	NADH:ubiquinone oxidoreductase core subunit V2	NDUFV2	64.22	12	12	−2.56	3.46E-02	
U3 J175	ATP synthase peripheral stalk-membrane subunit b	ATP5PB	45.61	15	15	−2.41	4.63E-03	
U3IK89	ATP synthase F1 subunit gamma	ATP5F1C	28.27	15	15	−2.40	4.90E-04	
U3I5C5	NADH dehydrogenase [ubiquinone] 1 alpha subcomplex subunit 9, mitochondrial	NDUFA9	48.81	16	16	−2.19	2.25E-02	FAD
U3J3L1	NADH:ubiquinone oxidoreductase core subunit V1	NDUFV1	47.16	11	11	−1.99	9.78E-03	FMN
U3I8R9	NADH dehydrogenase [ubiquinone] 1 alpha subcomplex subunit 10, mitochondrial	NDUFA10	36.34	11	11	−1.96	5.89E-03	FAD
U3 J741	NADH dehydrogenase [ubiquinone] 1 alpha subcomplex subunit 12	NDUFA12	65.63	7	7	−1.77	4.88E-02	
U3J1J0	Acyl-CoA dehydrogenase family member 9	ACAD9	60.22	33	33	−1.73	4.14E-04	FAD
U3I342	Ubiquinol-cytochrome c reductase core protein 2	UQCRC2	61.49	26	26	−1.71	4.82E-03	
U3IMS0	NADH:ubiquinone oxidoreductase core subunit S3	NDUFS3	55.45	13	13	−1.56	9.70E-03	

*TCA* tricarboxylic acid, *ETC* electron transport chain, *FMN* flavin mononucleotide, *FAD* flavin adenine dinucleotide

*Fold change is expressed as the ratio of the riboflavin-deficient to the control group. For diminished proteins, the fold change was transformed to the corresponding negative value

Of the proteins in the enriched TCA cycle, six proteins were downregulated (SDHA, succinate dehydrogenase [ubiquinone] iron-sulfur subunit (SDHB), DLD, succinate--CoA ligase [ADP/GDP-forming] subunit alpha (SUCLG1), dihydrolipoamide S-succinyltransferase (DLST), and fumarate hydratase (FH)), while one protein was upregulated (isocitrate dehydrogenase [NAD] subunit, mitochondrial (IDH3A)). Sixteen proteins were involved in fatty acid beta-oxidation (carnitine O-palmitoyltransferase 1 (CPT1A), carnitine palmitoyltransferase 2 (CPT2), DLD, ETFDH, long-chain-fatty-acid--CoA ligase 1 (ACSL1), ACADL, long-chain-fatty-acid--CoA ligase 5 (ACSL5), solute carrier family 25 member 20 (SLC25A20), ETFA, ACADS, 2,4-dienoyl-CoA reductase (DECR1), trifunctional enzyme subunit alpha (HADHA), carnitine palmitoyltransferase 1B (CPT1B), ACAD9, enoyl-CoA delta isomerase 1 (ECI1), and carnitine O-acetyltransferase (CRAT)), which were all diminished in the RD group. Fourteen proteins were involved in the electron transport chain (SDHA, SDHB, NADH-ubiquinone oxidoreductase 75 kDa subunit (NDUFS1), ATP synthase subunit O (ATP5PO), NADH:ubiquinone oxidoreductase core subunit V2 (NDUFV2), ATP synthase peripheral stalk-membrane subunit b (ATP5PB), ATP synthase F1 subunit gamma (ATP5F1C), NDUFA9, NDUFV1, NDUFA10, NADH dehydrogenase [ubiquinone] 1 alpha subcomplex subunit 12 (NDUFA12), ACAD9, Ubiquinol-cytochrome c reductase core protein 2 (UQCRC2), and NADH:ubiquinone oxidoreductase core subunit S3 (NDUFS3)), which were all diminished in the RD group.

## Discussion

Previous studies described inadequate riboflavin in the diet of laying hens resulted in low egg hatchability and abnormal embryonic development [5, 6]. Similarly, inherited RD chicken embryos presented delayed development, fatty livers, and extensive cutaneous hemorrhaging, and suddenly death at mid-incubation [8]. Recently, Cogburn et al. (2018) showed that RD in mid-stage embryos leads to the reduced expression of numerous genes involved in the beta-oxidation of lipids and energy depletion at the transcriptional level [8]. However, limited data are available currently at the protein level-the functional relevant integration level concerning the molecular mechanisms behind induced by maternal RD. The aim of this study was to investigate the underlying mechanisms of embryonic death induced by maternal RD using a proteomic approach. Proteomic analysis revealed that maternal RD mainly affected proteins involved in the TCA cycle, fatty acid beta-oxidation, and ETC processes, suggesting that these processes are associated with embryonic death. Notably, all flavoproteins or subunits of flavin-dependent enzymes among the RD altered proteins, such as DLD, SDHA, ETFDH, ACADS, ACADL, ETFA, NDUFA9, NDUFV1, NDUFA10, and ACAD9, were greatly reduced in the liver from RD embryos. This finding is consistent with the hypothesis that flavoprotein expression may be downregulated due to a reduced supply of riboflavin in the diet and is in line with previous animal and human studies [8, 20, 21].

The riboflavin concentrations in maternal plasma and egg yolk dropped dramatically in the RD group, indicating that these ducks were riboflavin deficient. The current study demonstrated that maternal diet deficient in riboflavin caused embryonic death in ducks, and the major peak of embryonic mortality was at the early stage of incubation. This finding confirms data from laying hens fed a diet with inadequate riboflavin showed depressed hatchability [5, 6], and is in accordance with the observation that the excessive depletion of riboflavin reserves in hens caused a major peak of embryonic mortality on the fourth day [22]. In the present study, the majority of RD embryos (73.2%) were nonviable within 13 days of incubation, confirming previous findings inherited RD chicken embryos suddenly died mid-incubation (days 13–15) [8]. Therefore, we used a proteomic approach to investigate the metabolic disorders of E13 embryonic liver tissue induced by maternal RD to explain embryonic death. Proteomics analysis revealed 589 differentially expressed proteins in the livers of RD embryos compared to those that were adequately supplied with riboflavin, indicating an important impact of riboflavin on embryonic development. The identical proteins are mainly enriched in the TCA cycle, fatty acid beta-oxidation, and ETC processes based on KEGG analysis.

RD downregulated six proteins involved in the TCA cycle, including DLD, SDHA, SDHB, SUCLG1, DLST, and FH, and upregulated one protein, IDH3A. The results obtained in previous human and animal studies [20, 21], showing that DLD was diminished in the skeletal muscle of riboflavin-responsive multiple acyl-CoA dehydrogenase deficiency (RR-MAD) patient and in RD ducks, consistent with our results. DLD (E3) is a common component of pyruvate and α-ketoglutarate dehydrogenase complexes, converting dihydrolipoic acid and NAD^+^ into lipoic acid and NADH [23]. Pyruvate dehydrogenase catalyses the oxidative decarboxylation of pyruvate to acetyl-CoA, thereby linking glycolysis to the TCA cycle and fatty acid synthesis [24, 25]. DLST (E2) is also a common component of α-ketoglutarate dehydrogenase, catalysing the conversion of α-ketoglutarate to succinyl-CoA and NADH, which is a rate-limiting enzyme of the TCA cycle [26, 27]. SDHA and SDHB are two subunits of succinate dehydrogenase complex, which catalyses the oxidation of succinate to fumarate [28]. SUCLG1 is a subunit of the heterodimeric enzyme succinate coenzyme A ligase, which catalyses the conversion of succinyl CoA and ADP or GDP to succinate and ATP or GTP [29]. FH catalyses the reversible hydration/dehydration of fumarate to malate [30]. IDH3A is a subunit of isocitrate dehydrogenase, which catalyzes the decarboxylation of isocitrate into alpha-ketoglutarate [31]. Six out of seven proteins (DLD, SDHA, SDHB, SUCLG1, DLST, and FH) were depressed in the RD group, which probably indicates a decreased liver TCA cycle.

RD downregulated 16 proteins involved in fatty acid beta-oxidation, including ETFA, ETFDH, CPT1A, CPT1B, CPT2, ACADS, ACAD9, ACADL, DLD, ACSL1, ACSL5, DECR1, SLC25A20, HADHA, ECI1, and CRAT. Previous studies showed that ACADS, ACAD9, and ETFDH were downregulated in the RD group in humans and animals, which is consistent with our results [20, 21]. CPT1A and CPT1B are two isoforms of carnitine palmitoyltransferase 1 (CPT1), an outer membrane protein that catalyses activated fatty acids into acylcarnitines, which is the first committed and regulated step in mitochondrial fatty acid oxidation [32, 33]. CPT2, an inner membrane protein, catalyses the formation of acyl-CoA from acylcarnitine and CoA [34]. CRAT catalyses the reversible transfer of acyl-CoA from carnitine to free CoA [35]. SLC25A20 facilitates the transfer of acylcarnitine esters in exchange for free carnitine across the mitochondrial membrane [36]. ACADS, ACADL, and ACAD9 belong to the family of fatty acyl-CoA dehydrogenases that catalyse the initial rate-limiting step of the beta-oxidation cycle [37]. HADHA converts medium- and long-chain 2-enoyl-CoA compounds into 3-ketoacyl-CoA. ETFA and ETFDH accept electrons from multiple acetyl-CoA dehydrogenases and subsequently transfer these to ETC [38]. ACSL1 and ACSL5 belong to the acyl-CoA synthetase family, which plays an important role in fatty acid catabolism and de novo lipid synthesis, catalysing the initial fatty acid activation by forming a thioester with CoA [39]. ECI1 and DECR1 have important roles in the metabolism of unsaturated fatty acids in beta oxidation. ECI1 is an auxiliary enzyme in the beta oxidation of unsaturated fatty acids that converts 3-cis or trans-enoyl-CoA to 2-trans-enoyl-CoA [40]. DECR1 participates in the metabolism of (poly)unsaturated fatty enoyl-CoA esters with double bonds in both even- and odd-numbered positions, and catalyses the NADP-dependent reduction of 2,4-dienoyl-CoA to yield trans-3-enoyl-CoA [41]. The decreased expression of these proteins involved in the fatty acid beta-oxidation process may imply that mitochondrial fatty acid beta-oxidation is impaired by RD. Furthermore, downregulation of these proteins involved in the fatty acid beta-oxidation process due to RD could very well explain the observed elevation of liver TG levels in E27 embryos. This explanation is supported by previous findings in humans and animals [9, 20, 21, 42–44].

RD downregulated 14 proteins involved in the ETC process, including SDHA, SDHB, NDUFS1, ATP5PO, NDUFV2, ATP5PB, ATP5F1C, NDUFA9, NDUFV1, NDUFA10, NDUFA12, ACAD9, UQCRC2, and NDUFS3. NDUFS1, NDUFS3, NDUFV1, NDUFV2, NDUFA9, NDUFA10, and NDUFA12 are seven subunits of complex I, which play a direct role in complex I assembly [45]. ACAD9 not only plays a physiological role in the beta oxidation of fatty acids, but also serves as an assembly factor for mitochondrial respiratory chain complex I [46]. The reduction of these proteins in RD embryos is in line with the role of riboflavin in complex I assembly [47]. As two subunits of complex II, SDHA and SDHB not only play an important role in the TCA cycle but also feed electrons to the respiratory chain ubiquinone pool [28]. A previous study showed that SDHA mutations caused a complex II deficiency [48]. UQCRC2 is a subunit of complex III, which is required for the assembly and stabilization of the complex [49]. ATP5PO, ATP5PB, and ATP5F1C are three subunits of complex V, which play a direct role in complex V assembly [50, 51]. The downregulated expression of proteins involved in the ETC process, including complex I, complex II, complex III, and complex V, likely indicates that mitochondrial oxidative phosphorylation is impaired by RD, which was supported by previous studies [8, 21, 44, 52].

Our proteomic analysis revealed an impairment of fatty acid beta-oxidation, TCA cycle, and ETC processes in RD embryos. Together, these impaired processes may lead to insufficient ATP production and subsequent embryonic death.

## Conclusions

Maternal RD causes embryonic death and abnormal development in ducks. Our analysis of embryonic liver proteomics provides the first global view of the protein level responses to maternal RD and illustrates the impairment of vital biological processes. Proteomic analysis showed that RD mainly diminished the expression of proteins involved in the TCA cycle, fatty acid beta-oxidation, and ETC processes in the livers of embryos, indicating that these processes were impaired and thus energy generation was reduced, which may lead to embryonic death. This finding adds to our understanding of the mechanisms underlying embryonic death as a result of maternal RD.

## Additional files

Additional file 1: Egg weight of ducks in the riboflavin-deficient (RD) group and the control (CON) group. (DOCX 14 kb)

Additional file 2: Egg production of ducks in the riboflavin-deficient (RD) group and the control (CON) group*. (DOCX 15 kb)*

Additional file 3: Egg fertility rate of ducks in the riboflavin-deficient (RD) group and the control (CON) group*. (DOCX 14 kb)*

Additional file 4: Differentially expressed proteins in E13 embryonic liver from maternal ducks after 8-week riboflavin depletion. (DOCX 108 kb)

## Abbreviations

CON: Control
ETC: Electron transport chain
FAD: Flavin adenine dinucleotide
FC: Fold change
FMN: Flavin mononucleotide
GO: Gene Ontology
HPLC: High performance liquid chromatography
iTRAQ: Isobaric tags for relative and absolute quantification
KEGG: Kyoto Encyclopedia of Genes and Genomes database
RD: Riboflavin deficiency
SEM: The standard error of the means
TCA: Tricarboxylic acid
TG: Triglyceride
