# Supplementary material for: Maternal diet deficient in riboflavin induces embryonic death associated with alterations in the hepatic proteome of duck embryos
Source: Nutr Metab (Lond). 2019 Mar 14;16:19. doi: 10.1186/s12986-019-0345-8 (PMC6419344; doi:10.1186/s12986-019-0345-8)
Supplement: Supplementary file 1 — Egg weight of ducks in the riboflavin-deficient (RD) group and the control (CON) group. (DOCX 14 kb) [file 12986_2019_345_MOESM1_ESM.docx]

Additional file 1. Egg weight of ducks in the riboflavin-deficient (RD) group and the control (CON) group.

| Time | RD (g) | CON (g) | SEM | *P*-value |
| --- | --- | --- | --- | --- |
| 1wk | 93.7 | 93.3 | 0.57 | 0.788 |
| 2wk | 92.2 | 93.0 | 0.61 | 0.940 |
| 3wk | 90.7 | 90.7 | 0.66 | 0.975 |
| 4wk | 91.1 | 91.5 | 0.65 | 0.407 |
| 5wk | 91.6 | 92.3 | 0.61 | 0.566 |
| 6wk | 90.6 | 91.8 | 0.63 | 0.342 |
| 7wk | 90.1 | 90.2 | 0.61 | 0.953 |
| 8wk | 90.0 | 90.0 | 0.61 | 0.999 |

SEM: standard error of the mean.
