# Supplementary material for: Maternal diet deficient in riboflavin induces embryonic death associated with alterations in the hepatic proteome of duck embryos
Source: Nutr Metab (Lond). 2019 Mar 14;16:19. doi: 10.1186/s12986-019-0345-8 (PMC6419344; doi:10.1186/s12986-019-0345-8)
Supplement: Supplementary file 2 — Egg production of ducks in the riboflavin-deficient (RD) group and the control (CON) group. (DOCX 15 kb) [file 12986_2019_345_MOESM2_ESM.docx]

Additional file 2. Egg production of ducks in the riboflavin-deficient (RD) group and the control (CON) group.

| Time | RD(%) | CON(%) | SEM | *P*-value |
| --- | --- | --- | --- | --- |
| 1wk | 95.6 | 93.7 | 1.15 | 0.516 |
| 2wk | 92.6 | 92.7 | 1.12 | 0.885 |
| 3wk | 89.1 | 92.7 | 1.62 | 0.225 |
| 4wk | 93.9 | 95.3 | 1.41 | 0.601 |
| 5wk | 93.2 | 93.7 | 1.38 | 0.920 |
| 6wk | 96.3 | 93.7 | 1.23 | 0.302 |
| 7wk | 91.7 | 93.7 | 1.82 | 0.776 |
| 8wk | 92.6 | 94.7 | 1.54 | 0.540 |

SEM: standard error of the mean.
