# Supplementary material for: Maternal diet deficient in riboflavin induces embryonic death associated with alterations in the hepatic proteome of duck embryos
Source: Nutr Metab (Lond). 2019 Mar 14;16:19. doi: 10.1186/s12986-019-0345-8 (PMC6419344; doi:10.1186/s12986-019-0345-8)
Supplement: Supplementary file 3 — Egg fertility rate of ducks in the riboflavin-deficient (RD) group and the control (CON) group. (DOCX 14 kb) [file 12986_2019_345_MOESM3_ESM.docx]

Additional file 3. Egg fertility rate of ducks in the riboflavin-deficient (RD) group and the control (CON) group.

| Time | RD (%) | CON (%) | SEM | *P*-value |
| --- | --- | --- | --- | --- |
| 1wk | 92.1 | 93.2 | 1.11 | 0.967 |
| 2wk | 92.0 | 93.7 | 1.04 | 0.468 |
| 3wk | 96.4 | 95.0 | 0.89 | 0.196 |
| 4wk | 91.5 | 95.5 | 1.57 | 0.259 |
| 5wk | 86.6 | 93.5 | 1.89 | 0.347 |
| 6wk | 85.3 | 91.0 | 1.80 | 0.207 |
| 7wk | 82.7 | 89.8 | 2.00 | 0.073 |
| 8wk | 76.4 | 83.7 | 2.63 | 0.187 |

SEM: standard error of the mean.
