# Supplementary material for: Maternal diet deficient in riboflavin induces embryonic death associated with alterations in the hepatic proteome of duck embryos
Source: Nutr Metab (Lond). 2019 Mar 14;16:19. doi: 10.1186/s12986-019-0345-8 (PMC6419344; doi:10.1186/s12986-019-0345-8)
Supplement: Supplementary file 4 — Differentially expressed proteins in E13 embryonic liver from maternal ducks after 8-week riboflavin depletion. (DOCX 108 kb) [file 12986_2019_345_MOESM4_ESM.docx]

Additional file 4. Differentially expressed proteins in E13 embryonic liver from maternal ducks after 8-week riboflavin depletion.

| UniProtKB ID | Protein description | Protein name | % coverage | Peptides number | Unique peptides | Fold change^*^ | *P*-Value |
| --- | --- | --- | --- | --- | --- | --- | --- |
| R0JU81 | Carboxylic ester hydrolase | Anapl_00395 | 49.82 | 28 | 4 | -14.91 | 4.41E-03 |
| U3JA11 | Uncharacterized protein | LOC101793408 | 25.22 | 36 | 36 | -13.24 | 9.93E-14 |
| R0KDM8 | Vitellogenin-1 | Anapl_04661 | 18.78 | 28 | 28 | -9.98 | 1.59E-04 |
| U3IWK0 | Uncharacterized protein | AADAC | 46.9 | 15 | 15 | -9.50 | 2.18E-09 |
| U3J7S6 | Uncharacterized protein | LOC101796596 | 48.61 | 20 | 15 | -9.06 | 3.70E-08 |
| R0J7H7 | Hemoglobin subunit epsilon | LOC101798290 | 78.91 | 23 | 12 | -7.48 | 3.97E-03 |
| U3J9A9 | 3-hydroxy-3-methylglutaryl coenzyme A synthase | HMGCS2 | 50.63 | 34 | 34 | -7.33 | 4.07E-09 |
| U3I6G0 | Uncharacterized protein | GATM | 33.89 | 14 | 14 | -6.99 | 6.80E-08 |
| R0LXL9 | Sulfotransferase | Anapl_15271 | 52.77 | 15 | 14 | -6.90 | 4.97E-07 |
| U3IJU8 | Aldehyde dehydrogenase 8 family member A1 | ALDH8A1 | 48.36 | 17 | 17 | -6.81 | 1.05E-07 |
| C7EKN9 | Hemoglobin alpha A subunit | HBA2 | 75.35 | 35 | 32 | -6.71 | 4.00E-05 |
| U3J2H8 | Fructose-bisphosphatase 1 | FBP1 | 66.21 | 42 | 42 | -6.04 | 2.52E-12 |
| U3J0A6 | Urocanate hydratase 1 | UROC1 | 32.89 | 24 | 24 | -6.02 | 1.77E-09 |
| U3J5N7 | Uncharacterized protein | LOC101800414 | 54.67 | 12 | 12 | -5.90 | 1.05E-05 |
| R0J775 | Aconitase 1 | ACO1 | 50.84 | 40 | 40 | -5.62 | 6.44E-15 |
| U3J3X9 | 5-aminoimidazole-4-carboxamide ribonucleotide formyltransferase/IMP cyclohydrolase | ATIC | 71.02 | 42 | 42 | -5.54 | 6.73E-14 |
| U3I256 | Dihydropyrimidinase | DPYS | 35.21 | 14 | 13 | -5.46 | 7.71E-05 |
| R0L018 | Carnitine O-palmitoyltransferase I, liver isoform | CPT1A | 46.1 | 35 | 35 | -5.45 | 2.11E-14 |
| R0K9G9 | 1-acyl-sn-glycerol-3-phosphate acyltransferase gamma | Anapl_03663 | 33.24 | 13 | 13 | -5.35 | 5.43E-06 |
| U3INM7 | Carnitine palmitoyltransferase 2 | CPT2 | 49.62 | 33 | 33 | -5.20 | 5.01E-09 |
| U3I2B5 | Uncharacterized protein | N/A | 38.04 | 20 | 19 | -5.18 | 1.50E-06 |
| U3IHF6 | Succinate dehydrogenase [ubiquinone] flavoprotein subunit, mitochondrial | SDHA | 32.23 | 17 | 17 | -5.17 | 2.24E-08 |
| R0LRR4 | Hydroxysteroid dehydrogenase-like protein 2 | Anapl_08159 | 54.98 | 25 | 25 | -5.06 | 7.70E-08 |
| U3J6P0 | Uncharacterized protein | C3 | 15.94 | 27 | 27 | -5.05 | 4.16E-10 |
| P13743 | L-lactate dehydrogenase B chain | LDHB | 74.47 | 38 | 37 | -5.02 | 2.10E-09 |
| R0LBD9 | Sulfotransferase | Anapl_16218 | 45.86 | 17 | 16 | -5.00 | 2.52E-05 |
| U3IGQ0 | Alanine--glyoxylate aminotransferase 2 | AGXT2 | 42.02 | 14 | 14 | -4.97 | 3.42E-06 |
| C7EMB6 | Hemoglobin beta A subunit | HBB | 81.63 | 32 | 17 | -4.88 | 3.44E-04 |
| U3ILQ9 | Uncharacterized protein | LOC101794959 | 38.36 | 16 | 16 | -4.84 | 4.65E-07 |
| R0L3F2 | Microsomal triglyceride transfer protein large subunit | Anapl_12362 | 48.45 | 37 | 37 | -4.81 | 9.89E-11 |
| U3J5X3 | Succinate dehydrogenase [ubiquinone] iron-sulfur subunit, mitochondrial | SDHB | 41.01 | 11 | 11 | -4.80 | 5.56E-05 |
| P04442 | Hemoglobin subunit alpha-D | HBAD | 84.4 | 25 | 23 | -4.74 | 4.95E-05 |
| U3J8P1 | Sulfotransferase | LOC101790335 | 35.35 | 11 | 10 | -4.74 | 1.49E-04 |
| U3IM27 | Uncharacterized protein | ALDH2 | 61.8 | 32 | 30 | -4.73 | 1.34E-07 |
| R0KYP5 | Hemoglobin subunit rho | Anapl_16678 | 75.51 | 27 | 9 | -4.71 | 1.27E-02 |
| U3IUY3 | Serine hydroxymethyltransferase | SHMT1 | 54.47 | 32 | 32 | -4.68 | 3.18E-07 |
| R0LA79 | D-dopachrome decarboxylase | DDT | 79.66 | 8 | 8 | -4.67 | 3.90E-04 |
| U3IEH8 | Acetyl-CoA acetyltransferase 2 | ACAT2 | 43.45 | 10 | 10 | -4.66 | 1.34E-03 |
| U3IWF6 | Uncharacterized protein | LOC101804020 | 38.14 | 10 | 10 | -4.62 | 2.13E-03 |
| R0JDK1 | Hemoglobin subunit pi | HBZ | 95.07 | 20 | 18 | -4.60 | 3.53E-06 |
| U3IU92 | Albumin | ALB | 78.31 | 54 | 54 | -4.59 | 0.00E+00 |
| U3IR48 | Dihydrolipoyl dehydrogenase | DLD | 42.38 | 18 | 18 | -4.54 | 1.91E-05 |
| U3IT84 | Glyoxylate and hydroxypyruvate reductase | GRHPR | 40.34 | 8 | 8 | -4.53 | 4.30E-04 |
| U3IR52 | Alpha-enolase | ENO1 | 79.72 | 46 | 46 | -4.52 | 1.78E-08 |
| U3IA01 | Hematopoietic prostaglandin D synthase | HPGDS | 49.01 | 15 | 14 | -4.49 | 8.90E-05 |
| U3J8D5 | Uncharacterized protein | LOC101796420 | 35.54 | 18 | 14 | -4.47 | 4.06E-05 |
| U3ITI1 | Fumarylacetoacetate hydrolase | FAH | 49.16 | 17 | 17 | -4.47 | 6.18E-06 |
| R0JV14 | Carboxylic ester hydrolase | Anapl_00396 | 29.07 | 13 | 13 | -4.45 | 5.02E-04 |
| U3IKU2 | Sterol carrier protein 2 | SCP2 | 52.43 | 30 | 30 | -4.45 | 9.63E-10 |
| U3J597 | Isocitrate dehydrogenase [NADP] | IDH1 | 66.51 | 33 | 33 | -4.44 | 1.87E-10 |
| U3IN97 | Adenylosuccinate lyase | ADSL | 41.2 | 19 | 19 | -4.40 | 3.94E-06 |
| U3IB67 | Serpin family C member 1 | SERPINC1 | 25.32 | 13 | 13 | -4.38 | 1.42E-04 |
| U3I3L3 | Phenazine biosynthesis like protein domain containing | PBLD | 35.99 | 10 | 10 | -4.36 | 2.32E-03 |
| R0JPE3 | C-1-tetrahydrofolate synthase, cytoplasmic | Anapl_09506 | 67.21 | 59 | 58 | -4.35 | 4.90E-13 |
| U3ILF5 | Phosphoglycerate kinase | PGK1 | 72.6 | 36 | 36 | -4.35 | 3.59E-10 |
| R0JZP2 | Microsomal glutathione S-transferase 1 | MGST1 | 30.19 | 6 | 6 | -4.34 | 3.98E-02 |
| R0LSV8 | Electron transfer flavoprotein-ubiquinone oxidoreductase, mitochondrial | ETFDH | 47.37 | 25 | 25 | -4.33 | 4.08E-09 |
| U3J8G8 | Formimidoyltransferase cyclodeaminase | FTCD | 51.01 | 27 | 27 | -4.33 | 8.01E-08 |
| U3IA23 | Fibrinogen gamma chain | FGG | 40 | 14 | 14 | -4.24 | 1.26E-04 |
| U3IU30 | Acyl-CoA synthetase long chain family member 1 | ACSL1 | 58.08 | 43 | 40 | -4.23 | 1.38E-09 |
| R0KXM3 | Succinate dehydrogenase cytochrome b560 subunit, mitochondrial | Anapl_18161 | 21.78 | 4 | 4 | -4.08 | 2.43E-02 |
| U3I9J4 | Cytochrome P450 family 27 subfamily A member 1 | CYP27A1 | 38.9 | 14 | 14 | -4.04 | 1.98E-06 |
| U3IPW5 | Uncharacterized protein | N/A | 7.115 | 4 | 4 | -3.98 | 3.55E-04 |
| U3IRQ2 | UDP-glucuronosyltransferase | LOC101796668 | 8.801 | 4 | 4 | -3.90 | 2.43E-02 |
| U3IKG5 | Aldehyde dehydrogenase 9 family member A1 | ALDH9A1 | 44.96 | 23 | 22 | -3.88 | 1.42E-04 |
| R0LKH5 | Estradiol 17-beta-dehydrogenase 2 | HSD17B2 | 29.49 | 10 | 10 | -3.85 | 5.81E-05 |
| R0JJ64 | Cytochrome P450 2H1 | LOC101797666 | 30.36 | 12 | 10 | -3.85 | 9.47E-04 |
| U3ILA4 | Prostaglandin reductase 1 | PTGR1 | 30.84 | 9 | 9 | -3.83 | 7.69E-05 |
| R0LET4 | Glycogen [starch] synthase | GYS2 | 11.65 | 8 | 8 | -3.83 | 5.17E-04 |
| U3IM00 | Dimethylglycine dehydrogenase | DMGDH | 49.03 | 35 | 35 | -3.77 | 1.18E-07 |
| R0K7N7 | Aspartate aminotransferase | Anapl_06784 | 45.76 | 11 | 10 | -3.72 | 7.07E-04 |
| U3I6Q7 | Uncharacterized protein | LOC101799420 | 66.67 | 16 | 16 | -3.71 | 5.98E-06 |
| R0JKM7 | Fetuin-B | Anapl_03192 | 17.84 | 5 | 5 | -3.68 | 1.71E-03 |
| R0JH14 | Glutamate dehydrogenase 1, mitochondrial | Anapl_04306 | 75.61 | 32 | 32 | -3.67 | 1.71E-08 |
| R0JDR4 | Pterin-4 alpha-carbinolamine dehydratase 1 | PCBD1 | 89.42 | 10 | 7 | -3.64 | 7.80E-04 |
| U3IAY7 | Acyl-CoA dehydrogenase long chain | ACADL | 50.7 | 26 | 26 | -3.57 | 4.72E-08 |
| R0L9C9 | Hepatic lectin | Anapl_10635 | 14.05 | 5 | 5 | -3.57 | 1.96E-03 |
| U3I8D8 | Triosephosphate isomerase | TPI1 | 87.2 | 22 | 22 | -3.55 | 9.36E-07 |
| U3J4T5 | S-formylglutathione hydrolase | ESD | 40 | 12 | 12 | -3.54 | 2.29E-03 |
| U3I4I8 | Uncharacterized protein | LOC101804666 | 42.13 | 14 | 14 | -3.53 | 1.21E-04 |
| U3IIU6 | 2,4-dienoyl-CoA reductase 2 | DECR2 | 36.9 | 10 | 10 | -3.50 | 1.69E-03 |
| U3I5K2 | Adipocyte plasma membrane associated protein | APMAP | 45.48 | 16 | 16 | -3.47 | 4.89E-06 |
| U3ISI9 | Transaldolase | TALDO1 | 44.26 | 14 | 14 | -3.45 | 3.39E-05 |
| U3J2F6 | Aminocarboxymuconate semialdehyde decarboxylase | ACMSD | 29.45 | 7 | 7 | -3.41 | 1.89E-03 |
| U3I742 | Uncharacterized protein | A2M | 26.71 | 29 | 28 | -3.32 | 6.85E-07 |
| U3J9N7 | Flavin-containing monooxygenase | FMO5 | 12.01 | 7 | 6 | -3.32 | 2.94E-02 |
| U3ICN0 | Mitochondrial pyruvate carrier | MPC2 | 32.81 | 5 | 5 | -3.32 | 2.06E-02 |
| U3J1B2 | Choline dehydrogenase | CHDH | 52.1 | 23 | 23 | -3.29 | 3.49E-07 |
| R0KBX6 | Protein disulfide-isomerase | PDIA4 | 46.34 | 38 | 36 | -3.28 | 8.23E-08 |
| U3J2X4 | Kynurenine 3-monooxygenase | KMO | 29.48 | 13 | 13 | -3.23 | 1.00E-04 |
| U3HYS6 | Threonine synthase like 2 | THNSL2 | 24.11 | 8 | 8 | -3.21 | 4.91E-04 |
| R0L5J2 | Zinc binding alcohol dehydrogenase domain containing 2 | ZADH2 | 37.7 | 11 | 11 | -3.19 | 1.30E-02 |
| U3J9R8 | Alpha-1,4 glucan phosphorylase | PYGL | 47.13 | 37 | 29 | -3.18 | 1.36E-07 |
| A0A172 | Catalase | CAT | 52.27 | 34 | 34 | -3.16 | 4.84E-10 |
| R0LD68 | Adenosylhomocysteinase | Anapl_02415 | 59.35 | 26 | 26 | -3.15 | 5.97E-06 |
| U3I386 | Glycerol-3-phosphate acyltransferase 1, mitochondrial | GPAM | 12.91 | 8 | 8 | -3.14 | 9.66E-05 |
| U3J5H0 | Chromosome 11 open reading frame 54 | C11orf54 | 46.06 | 11 | 11 | -3.13 | 1.09E-03 |
| R0JRK5 | Arylacetamide deacetylase-like 4 | Anapl_03399 | 14.8 | 5 | 5 | -3.12 | 2.18E-02 |
| U3IKH5 | Agmatinase | AGMAT | 21.31 | 6 | 6 | -3.11 | 2.19E-02 |
| U3IFC3 | Mitochondrial amidoxime reducing component 2 | MARC2 | 60.94 | 13 | 13 | -3.10 | 1.25E-03 |
| U3III1 | Acyl-CoA thioesterase 6 | ACOT6 | 33.25 | 11 | 11 | -3.10 | 2.08E-03 |
| R0L1N4 | Gamma-butyrobetaine dioxygenase | BBOX1 | 53.61 | 19 | 19 | -3.09 | 8.90E-05 |
| U3J9C7 | Solute carrier family 25 member 1 | SLC25A1 | 41.18 | 12 | 12 | -3.09 | 1.86E-04 |
| U3IB12 | Sorbitol dehydrogenase | SORD | 37.5 | 12 | 12 | -3.08 | 1.14E-03 |
| U3IYQ3 | Amine oxidase | MAOB | 19.81 | 8 | 7 | -3.07 | 3.37E-03 |
| R0LHU9 | Alpha-1-antiproteinase | Anapl_03302 | 25.18 | 9 | 9 | -3.07 | 1.35E-04 |
| R0JQZ8 | Acyl-coenzyme A thioesterase 4 | Anapl_15305 | 59.75 | 10 | 10 | -3.06 | 6.03E-04 |
| U3J4Z9 | Acyl-CoA synthetase long chain family member 5 | ACSL5 | 40.85 | 24 | 21 | -3.05 | 7.07E-07 |
| U3IGV1 | Glycerol-3-phosphate dehydrogenase [NAD(+)] | GPD1 | 61.36 | 18 | 16 | -3.02 | 3.17E-04 |
| U3IDJ3 | 4-hydroxyphenylpyruvate dioxygenase | HPD | 61.22 | 21 | 21 | -2.98 | 1.48E-04 |
| U3IC18 | Glutathione S-transferase kappa | GSTK1 | 54.87 | 12 | 12 | -2.97 | 1.97E-05 |
| R0LYU3 | Vitamin D-binding protein | Anapl_10497 | 17.89 | 9 | 9 | -2.94 | 1.36E-03 |
| A0A2H4 | Heat shock protein 90 kDa beta | Grp94 | 48.55 | 42 | 41 | -2.91 | 8.97E-07 |
| R0JK78 | Phosphate carrier protein, mitochondrial | SLC25A3 | 31.07 | 12 | 11 | -2.91 | 8.81E-03 |
| U3IFL0 | Solute carrier family 25 member 20 | SLC25A20 | 38.01 | 9 | 9 | -2.90 | 4.27E-04 |
| U3IR67 | Hydroxysteroid 17-beta dehydrogenase 4 | HSD17B4 | 44.05 | 30 | 30 | -2.89 | 1.26E-04 |
| U3IB00 | D-amino acid oxidase | DAO | 16.21 | 6 | 6 | -2.88 | 2.00E-02 |
| R0KQM7 | Dehydrogenase/reductase SDR family member 7 | Anapl_14408 | 59.38 | 19 | 19 | -2.88 | 2.29E-06 |
| U3ISF5 | Glutamine synthetase | GLUL | 27.3 | 10 | 10 | -2.87 | 5.41E-08 |
| R0JVQ4 | Dolichyl-diphosphooligosaccharide--protein glycosyltransferase subunit STT3A | Anapl_03984 | 11.49 | 9 | 6 | -2.87 | 3.90E-02 |
| U3ID96 | Nudix hydrolase 5 | NUDT5 | 56.82 | 16 | 16 | -2.87 | 6.29E-05 |
| R0JYP4 | Dolichyl-diphosphooligosaccharide--protein glycosyltransferase subunit STT3B | Anapl_06234 | 12.3 | 9 | 6 | -2.85 | 7.04E-03 |
| U3IF09 | Acetyl-CoA acyltransferase 1 | ACAA1 | 48.35 | 15 | 15 | -2.83 | 7.78E-04 |
| U3J540 | Uncharacterized protein | N/A | 39.57 | 11 | 10 | -2.81 | 1.78E-03 |
| U3IP37 | Apoptosis inducing factor mitochondria associated 1 | AIFM1 | 29.66 | 15 | 15 | -2.80 | 2.51E-03 |
| R0K4R7 | Delta-1-pyrroline-5-carboxylate dehydrogenase, mitochondrial | Anapl_04364 | 47.13 | 22 | 22 | -2.80 | 1.25E-04 |
| U3J802 | Spectrin beta, erythrocytic | SPTB | 6.936 | 10 | 7 | -2.79 | 1.34E-02 |
| R0K082 | NADH-ubiquinone oxidoreductase 75 kDa subunit, mitochondrial | NDUFS1 | 55.59 | 34 | 34 | -2.79 | 1.02E-06 |
| U3IMJ5 | Beta-ureidopropionase 1 | UPB1 | 40.38 | 14 | 14 | -2.79 | 2.63E-04 |
| U3ITZ1 | Cytochrome P450 | LOC101790812 | 24.95 | 10 | 8 | -2.78 | 2.83E-03 |
| R0K776 | Monocarboxylate transporter 1 | SLC16A1 | 15.43 | 7 | 7 | -2.78 | 4.20E-02 |
| U3IXR8 | Uncharacterized protein | LOC101802453 | 39.95 | 14 | 14 | -2.78 | 5.66E-05 |
| U3J2R0 | Vitronectin | VTN | 11.41 | 5 | 5 | -2.76 | 3.36E-02 |
| R0JSX9 | Fibrinogen alpha chain | Anapl_07733 | 16.87 | 9 | 9 | -2.75 | 1.85E-03 |
| R0JV77 | Sulfide:quinone oxidoreductase, mitochondrial | SQOR | 31.56 | 12 | 12 | -2.74 | 3.48E-03 |
| U3J6J0 | Succinate--CoA ligase [ADP/GDP-forming] subunit alpha, mitochondrial | SUCLG1 | 36.34 | 7 | 7 | -2.74 | 9.55E-03 |
| R0LHZ1 | 3-ketoacyl-CoA thiolase, mitochondrial | ACAA2 | 68.26 | 28 | 28 | -2.74 | 1.09E-03 |
| U3J4P9 | Ribosomal protein L3 | RPL3 | 43.42 | 20 | 20 | -2.73 | 1.12E-05 |
| U3IGY5 | Aspartate aminotransferase | GOT2 | 74.47 | 34 | 34 | -2.71 | 1.94E-07 |
| U3I2M8 | 3-hydroxybutyrate dehydrogenase 2 | BDH2 | 36.18 | 9 | 9 | -2.70 | 4.37E-03 |
| R0JS80 | Fibrinogen beta chain | Anapl_07732 | 46.05 | 17 | 17 | -2.69 | 3.84E-04 |
| U3J7F4 | Electron transfer flavoprotein alpha subunit | ETFA | 62.87 | 17 | 17 | -2.69 | 1.47E-02 |
| R0JZB9 | APOBEC1 complementation factor | A1CF | 33.96 | 15 | 14 | -2.69 | 7.23E-03 |
| U3J5E2 | S-(hydroxymethyl)glutathione dehydrogenase | ADH5 | 38.83 | 13 | 12 | -2.65 | 3.59E-03 |
| U3IWA6 | 3-hydroxyanthranilate 3,4-dioxygenase | HAAO | 57.64 | 14 | 14 | -2.64 | 2.89E-05 |
| U3J4P4 | Uncharacterized protein | SEC14L2 | 46.07 | 12 | 12 | -2.63 | 2.64E-03 |
| U3J4V2 | Tubulin beta chain | TUBB1 | 49.11 | 23 | 10 | -2.62 | 2.07E-02 |
| U3IFX9 | Kininogen 1 | KNG1 | 9.562 | 4 | 4 | -2.61 | 1.36E-02 |
| U3IF32 | Ankyrin 1 | ANK1 | 10.54 | 17 | 15 | -2.61 | 1.05E-04 |
| U3ILD0 | Uncharacterized protein | LOC101802407 | 58.52 | 38 | 38 | -2.61 | 5.55E-09 |
| U3J8V5 | MAM domain containing 4 | MAMDC4 | 12.56 | 12 | 12 | -2.61 | 8.41E-05 |
| U3J383 | Phosphoglucomutase 1 | PGM1 | 46.64 | 24 | 23 | -2.59 | 8.93E-06 |
| R0KV25 | Hepatic triacylglycerol lipase | Anapl_06158 | 17.91 | 5 | 5 | -2.58 | 1.71E-02 |
| U3IIA3 | Uncharacterized protein | DHFR | 60.25 | 10 | 10 | -2.58 | 1.24E-04 |
| R0LFF0 | Prostaglandin E synthase 2 | PTGES2 | 33.21 | 8 | 8 | -2.56 | 1.32E-02 |
| R0LIL9 | ATP synthase subunit O, mitochondrial | ATP5PO | 69.02 | 13 | 13 | -2.56 | 4.03E-03 |
| U3IKH0 | NADH:ubiquinone oxidoreductase core subunit V2 | NDUFV2 | 64.22 | 12 | 12 | -2.56 | 3.46E-02 |
| U3ID33 | NADPH--cytochrome P450 reductase | POR | 28.29 | 15 | 15 | -2.56 | 3.88E-03 |
| A0A172 | Superoxide dismutase [Cu-Zn] | SOD1 | 82.47 | 10 | 10 | -2.53 | 4.29E-02 |
| R0LS85 | Putative 2-oxoglutarate dehydrogenase E1 component DHKTD1, mitochondrial | Anapl_05882 | 43.3 | 25 | 25 | -2.52 | 1.25E-05 |
| R0LKA3 | 3-hydroxymethyl-3-methylglutaryl-CoA lyase | HMGCL | 43.39 | 11 | 11 | -2.52 | 3.02E-02 |
| U3IHZ7 | Delta-aminolevulinic acid dehydratase | ALAD | 52.99 | 11 | 11 | -2.51 | 9.30E-04 |
| U3IN13 | Uncharacterized protein | LOC101793912 | 36.31 | 12 | 9 | -2.51 | 7.23E-03 |
| U3J746 | RNA 3'-terminal phosphate cyclase | RTCA | 48.16 | 12 | 12 | -2.51 | 4.81E-05 |
| U3I8Z4 | Uncharacterized protein | N/A | 10.77 | 8 | 8 | -2.50 | 4.34E-03 |
| U3IVZ0 | Nitrilase family member 2 | NIT2 | 54.8 | 12 | 11 | -2.50 | 3.72E-04 |
| U3J485 | ATP binding cassette subfamily A member 1 | ABCA1 | 4.688 | 9 | 9 | -2.49 | 1.17E-02 |
| R0LAT5 | Protein disulfide-isomerase A5 | PDIA5 | 43.08 | 18 | 18 | -2.48 | 1.31E-03 |
| U3IHE6 | Uncharacterized protein | LOC101800188 | 4.425 | 6 | 6 | -2.46 | 2.10E-02 |
| U3I3S5 | Nicotinamide nucleotide transhydrogenase | NNT | 38.17 | 39 | 39 | -2.46 | 1.43E-07 |
| U3IX93 | High density lipoprotein binding protein | HDLBP | 40.33 | 47 | 47 | -2.46 | 3.50E-08 |
| R0K2Z3 | GDH/6PGL endoplasmic bifunctional protein | Anapl_11912 | 27.33 | 18 | 17 | -2.42 | 2.40E-03 |
| U3IX91 | Dihydropyrimidine dehydrogenase [NADP(+)] | DPYD | 18.05 | 11 | 11 | -2.42 | 1.16E-02 |
| U3J7B8 | Alpha-1-microglobulin/bikunin precursor | AMBP | 27.76 | 7 | 7 | -2.41 | 5.02E-03 |
| U3J175 | ATP synthase peripheral stalk-membrane subunit b | ATP5PB | 45.61 | 15 | 15 | -2.41 | 4.63E-03 |
| R0M413 | Pterin-4-alpha-carbinolamine dehydratase 2 | PCBD2 | 82.35 | 10 | 8 | -2.41 | 8.99E-03 |
| U3IK89 | ATP synthase F1 subunit gamma | ATP5F1C | 28.27 | 15 | 15 | -2.40 | 4.98E-04 |
| U3IYI1 | Uncharacterized protein | N/A | 46.15 | 13 | 13 | -2.40 | 5.15E-03 |
| U3ITR2 | Uncharacterized protein | LOC101799835 | 29.88 | 7 | 7 | -2.39 | 2.18E-02 |
| U3J8W0 | Acyl-CoA dehydrogenase short chain | ACADS | 49.3 | 14 | 14 | -2.36 | 4.32E-03 |
| U3IEM6 | Uncharacterized protein | LOC101804496 | 15.87 | 7 | 6 | -2.35 | 1.40E-02 |
| U3IST0 | Uncharacterized protein | N/A | 27.92 | 6 | 6 | -2.35 | 3.12E-02 |
| R0LQU7 | Adenosine kinase | ADK | 33.43 | 11 | 11 | -2.34 | 1.69E-03 |
| U3IRP7 | Solute carrier family 2 member 2 | SLC2A2 | 4.774 | 2 | 2 | -2.33 | 4.92E-02 |
| U3IP65 | Uncharacterized protein | N/A | 71.63 | 12 | 12 | -2.32 | 8.58E-03 |
| R0LI37 | Serine/threonine-protein kinase TOR | Anapl_03373 | 6.558 | 13 | 13 | -2.32 | 6.35E-03 |
| U3IFB0 | 2,4-dienoyl-CoA reductase 1 | DECR1 | 39.2 | 9 | 9 | -2.32 | 2.32E-02 |
| U3INV9 | ATP binding cassette subfamily D member 3 | ABCD3 | 19.27 | 11 | 11 | -2.31 | 2.20E-03 |
| U3J4H0 | Trans-L-3-hydroxyproline dehydratase | L3HYPDH | 31.22 | 6 | 6 | -2.30 | 2.27E-02 |
| U3IAV5 | Transmembrane protein 70 | TMEM70 | 20.11 | 4 | 4 | -2.30 | 2.96E-02 |
| U3IVL6 | Uncharacterized protein | N/A | 64.4 | 46 | 15 | -2.30 | 1.71E-02 |
| U3J8B9 | Angiotensinogen | AGT | 23.06 | 11 | 11 | -2.30 | 3.53E-03 |
| U3J3J8 | Glutathione S-transferase zeta 1 | GSTZ1 | 74.88 | 14 | 14 | -2.29 | 1.30E-03 |
| U3IXN1 | Peptidase M20 domain containing 1 | PM20D1 | 15.69 | 6 | 6 | -2.29 | 2.54E-02 |
| U3IMD9 | Nudix hydrolase 16 like 1 | NUDT16L1 | 55 | 9 | 9 | -2.29 | 1.08E-03 |
| R0JR75 | Trans-aconitate 3-methyltransferase | LOC101795599 | 29.26 | 8 | 8 | -2.28 | 1.05E-02 |
| U3J698 | Transketolase | TKT | 51.18 | 27 | 27 | -2.27 | 5.61E-05 |
| U3I046 | Glutathione peroxidase 1 | GPX1 | 58.97 | 8 | 8 | -2.27 | 3.61E-02 |
| U3IUA0 | Phenylalanine hydroxylase | PAH | 39.24 | 16 | 16 | -2.27 | 3.26E-03 |
| R0JWK3 | Dihydrolipoamide acetyltransferase component of pyruvate dehydrogenase complex | DBT | 46.43 | 17 | 16 | -2.26 | 1.76E-02 |
| R0KA30 | Mitochondrial dicarboxylate carrier | Anapl_02740 | 34.97 | 10 | 10 | -2.26 | 5.87E-04 |
| U3J928 | Acyl-coenzyme A oxidase | ACOX1 | 53.91 | 26 | 26 | -2.25 | 1.23E-05 |
| U3IHG8 | Fructose-bisphosphate aldolase | ALDOB | 89.29 | 83 | 39 | -2.25 | 6.63E-04 |
| U3J6I8 | Ferrochelatase | FECH | 41.54 | 13 | 13 | -2.25 | 2.90E-03 |
| U3IQB4 | Hydroxyacid oxidase 2 | HAO2 | 32.17 | 9 | 9 | -2.24 | 1.83E-02 |
| U3IXT4 | Methylmalonic aciduria (cobalamin deficiency) cblA type | MMAA | 14.43 | 5 | 5 | -2.24 | 1.32E-02 |
| U3ICH0 | Heat shock protein family E (Hsp10) member 1 | HSPE1 | 77.45 | 10 | 10 | -2.22 | 2.86E-02 |
| U3J562 | Phosphate cytidylyltransferase 2, ethanolamine | PCYT2 | 12.01 | 4 | 4 | -2.21 | 3.27E-02 |
| U3I7E1 | Sphingosine-1-phosphate lyase 1 | SGPL1 | 13.75 | 7 | 7 | -2.20 | 3.00E-02 |
| R0JVE7 | Peroxisomal bifunctional enzyme | Anapl_07826 | 50.67 | 31 | 19 | -2.20 | 1.85E-03 |
| R0LMP7 | Monoglyceride lipase | Anapl_08399 | 52.15 | 11 | 11 | -2.20 | 1.10E-02 |
| U3IV19 | Serpin family F member 2 | SERPINF2 | 12.47 | 5 | 5 | -2.19 | 3.05E-02 |
| U3I5C5 | Uncharacterized protein | NDUFA9 | 48.81 | 16 | 16 | -2.19 | 2.25E-02 |
| U3I9L4 | Dolichyl-diphosphooligosaccharide--protein glycosyltransferase subunit 1 | RPN1 | 44.01 | 25 | 24 | -2.19 | 4.24E-04 |
| U3I2N1 | Transglutaminase 2 | TGM2 | 20.77 | 11 | 11 | -2.19 | 6.45E-03 |
| U3J6U8 | Glycine C-acetyltransferase | GCAT | 57.14 | 18 | 18 | -2.18 | 4.48E-06 |
| U3IA87 | Serpin family D member 1 | SERPIND1 | 15.16 | 7 | 7 | -2.16 | 2.95E-03 |
| U3IQ07 | Uncharacterized protein | SCARB2 | 26.89 | 9 | 9 | -2.16 | 7.00E-03 |
| R0M765 | Elongation factor 1-alpha | EEF1A1 | 69.26 | 38 | 25 | -2.16 | 5.16E-03 |
| R0M006 | Alpha-aminoadipic semialdehyde synthase, mitochondrial | Anapl_12608 | 35.42 | 23 | 23 | -2.15 | 3.50E-03 |
| U3HZD9 | Regulator of microtubule dynamics 1 | RMDN1 | 39.08 | 10 | 10 | -2.15 | 4.83E-02 |
| U3INJ5 | 40S ribosomal protein S8 | RPS8 | 54.81 | 10 | 10 | -2.15 | 5.40E-03 |
| R0JD94 | 3-ketodihydrosphingosine reductase | KDSR | 26.69 | 6 | 6 | -2.14 | 1.87E-02 |
| U3IJE1 | Quinoid dihydropteridine reductase | QDPR | 53.31 | 7 | 7 | -2.13 | 1.70E-02 |
| U3IGC1 | SEC24 homolog C, COPII coat complex component | SEC24C | 26.4 | 22 | 22 | -2.12 | 1.96E-04 |
| U3I9L2 | Uncharacterized protein | N/A | 22.33 | 11 | 9 | -2.12 | 4.43E-02 |
| U3IJJ2 | Perilipin | N/A | 10.85 | 4 | 4 | -2.11 | 4.78E-02 |
| U3INW1 | Sideroflexin | SFXN1 | 41.98 | 12 | 11 | -2.11 | 8.97E-03 |
| U3J057 | Phosphatidylethanolamine binding protein 1 | PEBP1 | 58.94 | 9 | 9 | -2.10 | 5.37E-03 |
| R0KGC0 | Iodotyrosine dehalogenase 1 | Anapl_05314 | 27.84 | 7 | 7 | -2.08 | 1.60E-02 |
| U3I7I1 | 2-hydroxyacyl-CoA lyase 1 | HACL1 | 21.44 | 9 | 9 | -2.08 | 1.93E-02 |
| U3J069 | NAD(P) dependent steroid dehydrogenase-like | NSDHL | 23.14 | 7 | 7 | -2.07 | 1.33E-02 |
| U3IUK6 | Uncharacterized protein | N/A | 40.77 | 9 | 7 | -2.06 | 2.15E-03 |
| R0L1B4 | Inter-alpha-trypsin inhibitor heavy chain H2 | Anapl_17214 | 20.48 | 13 | 13 | -2.06 | 1.70E-03 |
| R0JE46 | Microsomal glutathione S-transferase 3 | MGST3 | 44.06 | 5 | 5 | -2.06 | 2.09E-02 |
| U3IRL7 | Uncharacterized protein | LOC101801727 | 49.89 | 19 | 19 | -2.06 | 5.46E-03 |
| U3IZX3 | Acyl-CoA thioesterase 13 | ACOT13 | 65.18 | 5 | 5 | -2.06 | 4.09E-02 |
| U3J8L3 | Solute carrier family 25 member 13 | SLC25A13 | 42.58 | 23 | 19 | -2.05 | 2.29E-05 |
| U3I806 | Uncharacterized protein | HADHA | 53.42 | 39 | 38 | -2.03 | 6.80E-03 |
| U3J4N1 | SAM and HD domain containing deoxynucleoside triphosphate triphosphohydrolase 1 | SAMHD1 | 18.16 | 6 | 6 | -2.03 | 5.45E-03 |
| U3IDQ1 | Acyl-coenzyme A oxidase | ACOX2 | 45.19 | 24 | 24 | -2.02 | 2.87E-03 |
| U3IZ26 | Disco interacting protein 2 homolog B | DIP2B | 5.11 | 5 | 5 | -2.02 | 3.36E-02 |
| R0JBZ4 | Coiled-coil domain containing 47 | CCDC47 | 18.67 | 8 | 8 | -2.02 | 3.14E-02 |
| U3IH78 | Dolichyl-diphosphooligosaccharide--protein glycosyltransferase 48 kDa subunit | DDOST | 30.24 | 8 | 8 | -2.01 | 2.50E-03 |
| U3IRP3 | Glucose-6-phosphate isomerase | GPI | 45.54 | 21 | 21 | -2.01 | 1.17E-04 |
| U3ITQ7 | Protein disulfide isomerase family A member 6 | PDIA6 | 27.25 | 11 | 11 | -2.01 | 4.45E-02 |
| U3J3L7 | Protein disulfide-isomerase | P4HB | 62.34 | 28 | 28 | -1.99 | 1.33E-03 |
| U3IIS8 | NME/NM23 nucleoside diphosphate kinase 4 | NME4 | 35 | 5 | 5 | -1.99 | 1.56E-02 |
| R0LKB1 | ATP-binding cassette sub-family B member 10, mitochondrial | Anapl_05252 | 2.044 | 1 | 1 | -1.99 | 2.12E-03 |
| U3J3L1 | NADH:ubiquinone oxidoreductase core subunit V1 | NDUFV1 | 47.16 | 11 | 11 | -1.99 | 9.79E-03 |
| U3INE4 | Carnosine dipeptidase 2 | CNDP2 | 39.96 | 17 | 17 | -1.98 | 1.11E-02 |
| R0LSD9 | Phosphotriesterase related | PTER | 32.66 | 11 | 11 | -1.98 | 1.32E-04 |
| R0KCX2 | Coagulation factor XIII A chain | F13A1 | 10.33 | 6 | 6 | -1.98 | 2.50E-03 |
| R0L951 | 3'(2'), 5'-bisphosphate nucleotidase 1 | BPNT1 | 40.58 | 10 | 10 | -1.97 | 1.86E-02 |
| U3INJ9 | ATP binding cassette subfamily C member 6 | ABCC6 | 4.668 | 5 | 5 | -1.96 | 4.81E-02 |
| U3I8R9 | NADH dehydrogenase [ubiquinone] 1 alpha subcomplex subunit 10, mitochondrial | NDUFA10 | 36.34 | 11 | 11 | -1.96 | 5.89E-03 |
| U3I632 | Calcium/calmodulin dependent protein kinase kinase 2 | CAMKK2 | 10.33 | 4 | 4 | -1.95 | 1.03E-02 |
| U3IZY1 | Aldehyde dehydrogenase 7 family member A1 | ALDH7A1 | 44.62 | 16 | 16 | -1.95 | 1.27E-02 |
| U3IB92 | Ribophorin II | RPN2 | 31.77 | 13 | 13 | -1.95 | 7.26E-04 |
| R0LSU2 | Plastin-3 | Anapl_04058 | 36.08 | 19 | 15 | -1.94 | 1.30E-03 |
| U3INY2 | Dihydrolipoamide S-succinyltransferase | DLST | 31.87 | 12 | 12 | -1.94 | 2.55E-02 |
| R0KYY1 | 60S ribosomal protein L18a | RPL18A | 39.62 | 7 | 7 | -1.94 | 2.16E-02 |
| U3IZ60 | Xylulokinase | XYLB | 28.6 | 10 | 10 | -1.94 | 1.87E-02 |
| R0KDW7 | Enoyl-CoA hydratase domain-containing protein 3, mitochondrial | Anapl_05876 | 42.39 | 9 | 9 | -1.93 | 1.95E-02 |
| U3IBD7 | Uncharacterized protein | LOC101789604 | 10.83 | 2 | 1 | -1.93 | 2.79E-02 |
| R0LB94 | Guanidinoacetate N-methyltransferase | Anapl_15096 | 47.33 | 8 | 8 | -1.92 | 7.26E-03 |
| R0JP74 | UDP-glucuronosyltransferase 1-1 | Anapl_12377 | 18.56 | 4 | 4 | -1.92 | 4.35E-02 |
| U3IB11 | Nicalin | NCLN | 23.63 | 9 | 9 | -1.90 | 4.13E-03 |
| U3IIV8 | Uncharacterized protein | CA5A | 24.28 | 5 | 5 | -1.90 | 3.71E-02 |
| U3IK00 | Carnitine palmitoyltransferase 1B | CPT1B | 17.83 | 7 | 7 | -1.89 | 2.53E-03 |
| U3IQT2 | ATP binding cassette subfamily B member 11 | ABCB11 | 5.232 | 5 | 5 | -1.88 | 3.80E-02 |
| U3IUY7 | Methylcrotonoyl-CoA carboxylase 2 | MCCC2 | 43.95 | 16 | 16 | -1.87 | 8.07E-03 |
| U3J925 | ATP-citrate synthase | ACLY | 44.14 | 45 | 45 | -1.87 | 5.58E-04 |
| R0LHJ8 | Clathrin heavy chain 1 | Anapl_03930 | 42.42 | 63 | 34 | -1.86 | 1.31E-03 |
| U3J4M7 | Membrane palmitoylated protein 6 | MPP6 | 19.07 | 9 | 9 | -1.86 | 2.46E-03 |
| R0M0V9 | Multifunctional protein ADE2 | Anapl_00811 | 64.05 | 26 | 26 | -1.86 | 1.49E-02 |
| R0JVP4 | Crystallin zeta | CRYZ | 38.37 | 10 | 10 | -1.85 | 2.70E-02 |
| U3II57 | Cytochrome P450 family 51 subfamily A member 1 | CYP51A1 | 7.039 | 3 | 3 | -1.85 | 4.42E-02 |
| U3ID73 | Uncharacterized protein | RPL7 | 54.47 | 18 | 17 | -1.84 | 4.96E-02 |
| U3IK61 | DNA helicase | MCM5 | 19.03 | 10 | 10 | -1.84 | 5.02E-03 |
| R0JMW7 | Fatty aldehyde dehydrogenase | Anapl_02624 | 37.95 | 16 | 15 | -1.83 | 3.80E-03 |
| U3I5P3 | Receptor protein-tyrosine kinase | EGFR | 15.25 | 16 | 16 | -1.83 | 4.28E-03 |
| U3J0R7 | Cytochrome P450 family 39 subfamily A member 1 | CYP39A1 | 7.234 | 3 | 3 | -1.82 | 4.66E-02 |
| R0LBE3 | Protein diaphanous-like protein 2 | Anapl_17291 | 9.687 | 10 | 10 | -1.82 | 4.90E-02 |
| U3IF99 | Branched chain keto acid dehydrogenase E1 subunit beta | BCKDHB | 45.65 | 9 | 8 | -1.82 | 2.92E-02 |
| U3J9U0 | Hydroxylysine kinase | HYKK | 15.84 | 5 | 5 | -1.81 | 2.16E-02 |
| U3J521 | Peptidylprolyl isomerase D | PPID | 36.68 | 14 | 13 | -1.81 | 1.29E-03 |
| R0L8I2 | Prohibitin | PHB | 65.82 | 17 | 17 | -1.81 | 2.02E-02 |
| U3J064 | Mitochondrial ribosomal protein L46 | MRPL46 | 28.02 | 5 | 5 | -1.81 | 3.36E-02 |
| U3IC24 | Kynurenine aminotransferase 1 | KYAT1 | 13.04 | 6 | 6 | -1.80 | 7.65E-03 |
| U3J6S8 | Uncharacterized protein | LOC101800887 | 14.43 | 6 | 6 | -1.80 | 4.36E-02 |
| R0KVC4 | Eukaryotic translation initiation factor 3 subunit C | Anapl_18626 | 20.98 | 6 | 6 | -1.80 | 3.63E-02 |
| U3IRU4 | ADP ribosylation factor guanine nucleotide exchange factor 2 | ARFGEF2 | 6.496 | 10 | 10 | -1.79 | 3.59E-03 |
| U3I593 | Receptor for activated C kinase 1 | RACK1 | 80.43 | 18 | 18 | -1.78 | 3.66E-03 |
| R0JC86 | 40S ribosomal protein S5 | Anapl_16687 | 41.33 | 8 | 8 | -1.78 | 2.60E-02 |
| R0K679 | Retinol-binding protein 4 | Anapl_03553 | 34.69 | 5 | 5 | -1.77 | 3.90E-02 |
| U3J741 | NADH dehydrogenase [ubiquinone] 1 alpha subcomplex subunit 12 | NDUFA12 | 65.63 | 7 | 7 | -1.77 | 4.88E-02 |
| U3IXU4 | Acyl-CoA-binding domain-containing protein 5 | ACBD5 | 14.04 | 5 | 5 | -1.76 | 2.30E-02 |
| U3IKM4 | Kynureninase | KYNU | 22.01 | 9 | 9 | -1.74 | 9.50E-03 |
| R0L1Y3 | 60 kDa heat shock protein, mitochondrial | HSPD1 | 76.61 | 59 | 59 | -1.74 | 2.76E-05 |
| U3IAX8 | Insulin degrading enzyme | IDE | 15.42 | 14 | 14 | -1.74 | 5.80E-03 |
| R0KC12 | Succinate-CoA ligase subunit beta | Anapl_03603 | 49.75 | 22 | 20 | -1.73 | 1.33E-02 |
| U3IRX8 | SEC31 homolog A, COPII coat complex component | SEC31A | 19.67 | 19 | 19 | -1.73 | 1.76E-02 |
| R0L6S9 | SRP receptor alpha subunit | SRPRA | 22.31 | 11 | 11 | -1.73 | 4.12E-02 |
| U3J1J0 | Acyl-CoA dehydrogenase family member 9 | ACAD9 | 60.22 | 33 | 33 | -1.73 | 4.14E-04 |
| U3IRP2 | Pyrophosphatase (inorganic) 2 | PPA2 | 33.02 | 9 | 9 | -1.72 | 2.49E-02 |
| U3J8P3 | Ribosomal protein L5 | RPL5 | 47.47 | 18 | 18 | -1.72 | 1.87E-02 |
| R0KJC4 | Alpha-mannosidase | Anapl_17796 | 24.15 | 20 | 20 | -1.72 | 9.13E-03 |
| R0JU52 | Proteasome-associated protein ECM29-like protein | Anapl_13317 | 6.155 | 9 | 9 | -1.71 | 1.65E-02 |
| U3I342 | Ubiquinol-cytochrome c reductase core protein 2 | UQCRC2 | 61.49 | 26 | 26 | -1.71 | 4.82E-03 |
| U3I7V0 | Ribosomal protein L24 | RPL24 | 30.57 | 6 | 6 | -1.71 | 4.79E-02 |
| U3I2B0 | 1,2-dihydroxy-3-keto-5-methylthiopentene dioxygenase | ADI1 | 39.55 | 5 | 5 | -1.71 | 2.42E-02 |
| U3J4H9 | Coenzyme Q8A | COQ8A | 13.08 | 7 | 7 | -1.70 | 1.64E-02 |
| R0K405 | Uncharacterized protein C7orf10 | Anapl_03265 | 20.54 | 5 | 5 | -1.69 | 2.44E-02 |
| R0KCV3 | Peroxiredoxin-4 | Anapl_12669 | 75.46 | 12 | 12 | -1.68 | 1.19E-02 |
| U3IJH1 | Mitochondrial ribosomal protein S7 | MRPS7 | 21.99 | 6 | 6 | -1.68 | 2.62E-02 |
| R0JXM5 | Malate dehydrogenase | MDH1 | 47.31 | 16 | 16 | -1.68 | 1.05E-02 |
| U3HZS2 | Crystallin mu | CRYM | 30.08 | 7 | 7 | -1.68 | 1.18E-02 |
| U3IBI2 | Elongation factor G, mitochondrial | GFM1 | 27.72 | 18 | 18 | -1.68 | 4.27E-03 |
| U3ILK4 | Uncharacterized protein | ECI1 | 50 | 11 | 11 | -1.67 | 1.13E-02 |
| U3IHS8 | Carnitine O-acetyltransferase | CRAT | 34.71 | 22 | 22 | -1.67 | 8.38E-04 |
| R0KT93 | Ubiquitin conjugation factor E4 B | Anapl_10891 | 4 | 5 | 5 | -1.67 | 1.66E-02 |
| U3J6F1 | Ribosomal protein L35a | RPL35A | 40.91 | 8 | 8 | -1.67 | 3.69E-02 |
| U3IQA4 | Uncharacterized protein | N/A | 60.23 | 20 | 6 | -1.66 | 3.23E-02 |
| U3J3I0 | DnaJ heat shock protein family (Hsp40) member A3 | DNAJA3 | 38.17 | 11 | 11 | -1.66 | 1.49E-02 |
| U3IGD1 | Adaptor related protein complex 2 mu 1 subunit | AP2M1 | 37.16 | 15 | 15 | -1.66 | 2.15E-02 |
| U3ITL1 | Eukaryotic translation elongation factor 1 gamma | EEF1G | 20.88 | 8 | 8 | -1.66 | 3.43E-02 |
| R0KBE2 | Fatty acid synthase | Anapl_02761 | 18.58 | 36 | 36 | -1.66 | 1.33E-04 |
| U3IWV6 | Uncharacterized protein | AFP | 44.39 | 30 | 30 | -1.65 | 3.92E-03 |
| U3J9R9 | AP-2 complex subunit alpha | AP2A2 | 36.43 | 29 | 28 | -1.65 | 2.27E-03 |
| U3I630 | Growth arrest specific 7 | GAS7 | 23.87 | 7 | 7 | -1.65 | 1.83E-02 |
| R0K7Y6 | 40S ribosomal protein S6 | RPS6 | 35.63 | 14 | 14 | -1.65 | 2.71E-02 |
| U3I4K0 | Uncharacterized protein | NIPSNAP3A | 23.48 | 6 | 6 | -1.64 | 1.51E-03 |
| R0K1H3 | Putative leucyl-tRNA synthetase, mitochondrial | Anapl_12821 | 10.73 | 8 | 8 | -1.64 | 4.83E-02 |
| R0JS40 | Putative eukaryotic translation initiation factor 3 subunit | Anapl_05839 | 29.33 | 33 | 32 | -1.64 | 6.98E-03 |
| U3IAB4 | Epithelial splicing regulatory protein 2 | ESRP2 | 15.19 | 8 | 8 | -1.63 | 1.20E-02 |
| L7XWS8 | Ferritin | FTH1 | 43.65 | 7 | 7 | -1.62 | 7.83E-03 |
| U3ILN2 | Ribosomal protein S2 | RPS2 | 58.55 | 15 | 15 | -1.62 | 1.36E-02 |
| U3IWC3 | Mitochondrial ribosomal protein L47 | MRPL47 | 22.12 | 5 | 5 | -1.62 | 1.77E-02 |
| R0L7Q0 | Fumarate hydratase | FH | 59.83 | 26 | 26 | -1.62 | 3.83E-03 |
| U3I6L2 | Lipase | LIPA | 6.329 | 3 | 3 | -1.62 | 2.46E-03 |
| U3I567 | Coatomer subunit beta | COPB2 | 43.97 | 31 | 31 | -1.61 | 1.71E-02 |
| R0JC25 | Ribosomal protein L15 | RPL15 | 33.98 | 7 | 7 | -1.60 | 2.44E-02 |
| U3IZ13 | Catechol-O-methyltransferase domain containing 1 | COMTD1 | 33.05 | 7 | 7 | -1.60 | 7.52E-03 |
| U3I7F8 | Uncharacterized protein | N/A | 51.12 | 8 | 8 | -1.60 | 3.93E-02 |
| U3I4P2 | SAMM50 sorting and assembly machinery component | SAMM50 | 43.13 | 18 | 18 | -1.60 | 5.45E-03 |
| R0L8K3 | 60S ribosomal protein L35 | RPL35 | 18.42 | 2 | 2 | -1.59 | 2.12E-02 |
| U3J4E5 | Ribosomal protein S3 | RPS3 | 55.06 | 15 | 15 | -1.58 | 2.47E-02 |
| U3IAV6 | Aldehyde dehydrogenase 1 family member A1 | ALDH1A1 | 50.31 | 25 | 22 | -1.57 | 3.20E-03 |
| U3I5D5 | Uncharacterized protein | NRDC | 11.64 | 10 | 10 | -1.57 | 1.51E-02 |
| R0KP25 | Calcium-binding protein 39 | CAB39 | 22.29 | 8 | 8 | -1.57 | 1.19E-02 |
| U3IMS0 | NADH:ubiquinone oxidoreductase core subunit S3 | NDUFS3 | 55.45 | 13 | 13 | -1.56 | 9.71E-03 |
| U3IJZ6 | Uncharacterized protein | LOC101794092 | 30.34 | 14 | 13 | -1.56 | 2.16E-02 |
| R0K9F9 | GMP synthase [glutamine-hydrolyzing] | GMPS | 38.19 | 12 | 12 | -1.56 | 3.47E-02 |
| R0L6N6 | Protein disulfide-isomerase | PDIA3 | 70.12 | 38 | 37 | -1.55 | 1.10E-03 |
| R0LG94 | Nucleobindin-2 | Anapl_14747 | 22.42 | 10 | 10 | -1.54 | 1.86E-03 |
| U3IC85 | Ribosomal protein L7a | RPL7A | 41.85 | 14 | 14 | -1.54 | 1.00E-02 |
| U3IH91 | Uncharacterized protein | N/A | 48.44 | 11 | 10 | -1.54 | 4.50E-02 |
| U3J6U6 | Kynurenine aminotransferase 3 | KYAT3 | 50.12 | 19 | 19 | -1.54 | 1.55E-02 |
| U3I826 | Tripeptidyl peptidase 2 | TPP2 | 22.82 | 23 | 23 | -1.53 | 9.68E-03 |
| R0K1Z3 | Superoxide dismutase | SOD | 40 | 8 | 8 | -1.53 | 1.97E-03 |
| U3IF19 | Eukaryotic translation initiation factor 3 subunit M | EIF3M | 22.93 | 7 | 7 | -1.53 | 2.14E-02 |
| U3IN86 | Methylcrotonoyl-CoA carboxylase 1 | MCCC1 | 30.35 | 16 | 14 | -1.52 | 3.18E-03 |
| R0KL06 | 39S ribosomal protein L15, mitochondrial | MRPL15 | 38.93 | 8 | 8 | -1.52 | 4.21E-02 |
| U3IBU3 | Prohibitin 2 | PHB2 | 53.68 | 11 | 11 | -1.51 | 1.93E-02 |
| U3IUW5 | DnaJ heat shock protein family (Hsp40) member C3 | DNAJC3 | 17.39 | 8 | 8 | -1.51 | 1.59E-02 |
| U3IYL2 | Seryl-tRNA synthetase | SARS | 42.67 | 15 | 15 | 1.50 | 1.89E-02 |
| R0J934 | Integrin alpha-V | Anapl_18005 | 12.6 | 7 | 7 | 1.50 | 2.66E-02 |
| R0LFS4 | Conserved oligomeric Golgi complex subunit 1 | Anapl_16453 | 2.908 | 2 | 2 | 1.51 | 3.77E-02 |
| U3J0G1 | Chaperonin containing TCP1 subunit 2 | CCT2 | 67.66 | 31 | 31 | 1.51 | 1.44E-02 |
| R0M4C8 | Ubiquitin carboxyl-terminal hydrolase 7 | Anapl_03972 | 9.898 | 9 | 9 | 1.51 | 3.45E-03 |
| U3IIB9 | Myosin heavy chain 9 | MYH9 | 43.89 | 82 | 57 | 1.51 | 8.24E-05 |
| U3IN65 | Plectin | PLEC | 11.15 | 41 | 41 | 1.52 | 6.15E-05 |
| U3IM52 | Protein kinase cAMP-dependent type I regulatory subunit alpha | PRKAR1A | 44.56 | 16 | 16 | 1.53 | 1.55E-02 |
| U3IWQ6 | Adenylyl cyclase-associated protein | CAP1 | 27.79 | 11 | 11 | 1.54 | 2.02E-02 |
| R0M1N7 | Cathepsin Z | CTSZ | 26.46 | 8 | 7 | 1.54 | 4.42E-02 |
| U3IJK0 | Myosin IC | MYO1C | 17.59 | 17 | 16 | 1.56 | 2.88E-03 |
| U3IJD5 | RNA cytidine acetyltransferase | NAT10 | 9.756 | 8 | 8 | 1.56 | 2.59E-02 |
| R0LB01 | Matrin-3 | Anapl_04954 | 31.49 | 24 | 24 | 1.56 | 3.02E-03 |
| U3J788 | ASPSCR1, UBX domain containing tether for SLC2A4 | ASPSCR1 | 28.35 | 9 | 9 | 1.57 | 4.94E-02 |
| R0LVN5 | Echinoderm microtubule-associated protein-like 4 | Anapl_01435 | 6.804 | 7 | 7 | 1.57 | 2.68E-03 |
| R0KY39 | Integrin linked kinase | ILK | 33.86 | 13 | 12 | 1.59 | 1.31E-02 |
| U3IUX2 | IQ motif containing GTPase activating protein 1 | IQGAP1 | 30.65 | 44 | 36 | 1.59 | 3.85E-04 |
| U3IEG9 | Far upstream element binding protein 1 | FUBP1 | 35.38 | 22 | 19 | 1.60 | 5.67E-03 |
| U3IF78 | Protein kinase cAMP-dependent type II regulatory subunit alpha | PRKAR2A | 35.86 | 8 | 7 | 1.61 | 4.77E-02 |
| R0JE49 | Eukaryotic translation initiation factor 4 gamma 3 | Anapl_13058 | 7.993 | 14 | 9 | 1.62 | 9.59E-03 |
| U3J5Y3 | Spectrin alpha, non-erythrocytic 1 | SPTAN1 | 46.06 | 102 | 101 | 1.62 | 3.51E-06 |
| U3IRD0 | Uncharacterized protein | N/A | 27.05 | 8 | 8 | 1.62 | 1.47E-02 |
| R0LKG6 | Cell division cycle 5-like protein | Anapl_01265 | 13.56 | 8 | 8 | 1.62 | 4.48E-02 |
| U3IAM7 | Golgi reassembly stacking protein 2 | GORASP2 | 22.09 | 6 | 6 | 1.63 | 3.14E-02 |
| U3J927 | Threonyl-tRNA synthetase | TARS | 31.88 | 21 | 16 | 1.63 | 2.52E-03 |
| R0JB17 | Phosphatidylinositol-binding clathrin assembly protein | Anapl_13686 | 8.333 | 4 | 4 | 1.63 | 4.37E-02 |
| A0A0D3RVN4 | Yolk sac IgY receptor | N/A | 3.379 | 4 | 4 | 1.63 | 4.13E-02 |
| U3J090 | Uncharacterized protein | HARS | 28.89 | 13 | 13 | 1.64 | 2.05E-02 |
| R0M8F5 | Conserved oligomeric Golgi complex subunit 4 | Anapl_07489 | 4.09 | 2 | 2 | 1.65 | 4.66E-02 |
| U3IIM5 | Leucine aminopeptidase 3 | LAP3 | 40.66 | 14 | 14 | 1.65 | 1.12E-02 |
| U3IND1 | Vesicle amine transport 1 like | VAT1L | 7.895 | 2 | 2 | 1.65 | 3.32E-03 |
| U3IGJ7 | Lactamase beta 2 | LACTB2 | 28.63 | 6 | 6 | 1.67 | 1.49E-02 |
| R0JS82 | Procollagen-lysine,2-oxoglutarate 5-dioxygenase 1 | Anapl_03388 | 16.79 | 10 | 9 | 1.67 | 7.00E-03 |
| U3IXQ0 | 3-hydroxyisobutyryl-CoA hydrolase, mitochondrial | HIBCH | 32.8 | 12 | 11 | 1.70 | 5.79E-03 |
| R0L6L8 | Cysteinyl-tRNA synthetase, cytoplasmic | Anapl_04133 | 27.68 | 20 | 20 | 1.70 | 3.45E-03 |
| R0L6X5 | 26S protease regulatory subunit 8 | PSMC5 | 62.01 | 20 | 18 | 1.70 | 2.80E-03 |
| U3IXS8 | Uncharacterized protein | LOC101798826 | 4.24 | 1 | 1 | 1.71 | 3.58E-02 |
| U3IKR7 | 10-formyltetrahydrofolate dehydrogenase | ALDH1L2 | 44.76 | 34 | 34 | 1.71 | 1.71E-03 |
| U3IPY8 | Uncharacterized protein | LOC101796074 | 30.49 | 35 | 34 | 1.72 | 2.00E-04 |
| R0M3E5 | Band 4.1-like protein 2 | Anapl_01837 | 13.31 | 11 | 10 | 1.72 | 8.04E-03 |
| U3IKT3 | Kinesin-like protein | KIF5B | 25.92 | 24 | 24 | 1.73 | 2.87E-03 |
| R0LQS4 | Calpain-1 catalytic subunit | Anapl_05703 | 15.89 | 9 | 9 | 1.73 | 5.34E-03 |
| R0JYN0 | Prostaglandin F2 receptor negative regulator | Anapl_01606 | 12.41 | 10 | 9 | 1.74 | 1.17E-03 |
| U3IIJ9 | Uncharacterized protein | GCLM | 30.74 | 7 | 7 | 1.74 | 3.86E-02 |
| U3IPR3 | Lamin A/C | LMNA | 46.81 | 32 | 30 | 1.74 | 6.49E-04 |
| R0LRM7 | Destrin | DSTN | 68.48 | 13 | 12 | 1.74 | 1.10E-02 |
| Q2PMX0 | GTP-binding protein | Rab | 61.06 | 11 | 10 | 1.75 | 4.75E-03 |
| U3IUN7 | Uncharacterized protein | RBMX | 28.57 | 11 | 11 | 1.75 | 1.19E-02 |
| U3IMW8 | Phosphatidylinositol transfer protein alpha | PITPNA | 30.37 | 7 | 4 | 1.78 | 3.01E-02 |
| U3I5V0 | Uncharacterized protein | LRRC47 | 23.75 | 8 | 8 | 1.78 | 2.89E-02 |
| U3IQJ8 | EPH receptor B3 | EPHB3 | 1.76 | 2 | 2 | 1.79 | 1.76E-02 |
| R0M3N3 | Coatomer subunit gamma-2 | Anapl_10463 | 16.39 | 11 | 9 | 1.79 | 4.46E-02 |
| U3I3N1 | Decapping enzyme, scavenger | DCPS | 12.73 | 4 | 4 | 1.80 | 3.57E-02 |
| U3J0C5 | GrpE protein homolog | GRPEL1 | 37.1 | 7 | 7 | 1.82 | 1.36E-02 |
| R0LCH9 | ATP-dependent metalloprotease YME1L1 | Anapl_14846 | 16.74 | 10 | 10 | 1.82 | 1.13E-02 |
| U3INH2 | Tropomodulin 3 | TMOD3 | 31.82 | 11 | 10 | 1.83 | 4.63E-03 |
| R0L4N0 | E3 ubiquitin-protein ligase HECTD1 | Anapl_11713 | 2.941 | 6 | 6 | 1.84 | 7.55E-03 |
| U3ICK7 | Phosphoserine aminotransferase | PSAT1 | 50.13 | 17 | 17 | 1.84 | 4.33E-04 |
| U3IQM6 | Septin 7 | SEPT7 | 44.58 | 14 | 14 | 1.84 | 1.16E-03 |
| A0A2D0 | Integrin beta | ITGB1 | 24.3 | 20 | 20 | 1.85 | 1.55E-03 |
| R0LS95 | DnaJ-like protein subfamily C member 10 | Anapl_00628 | 7.323 | 5 | 5 | 1.85 | 2.48E-02 |
| R0LDH3 | EH domain containing 3 | EHD3 | 48.6 | 25 | 21 | 1.85 | 2.36E-02 |
| U3IUS7 | NSFL1 cofactor | NSFL1C | 38.55 | 12 | 12 | 1.86 | 1.33E-02 |
| R0JJP0 | 5'-nucleotidase domain-containing protein 2 | Anapl_05040 | 18.43 | 9 | 9 | 1.86 | 9.14E-04 |
| U3INH3 | Sorbin and SH3 domain containing 2 | SORBS2 | 0.9892 | 1 | 1 | 1.87 | 2.85E-02 |
| U3IZF2 | Translocated promoter region, nuclear basket protein | TPR | 15.36 | 31 | 31 | 1.87 | 4.18E-04 |
| R0K2I4 | Lon protease homolog | Anapl_10871 | 38.73 | 29 | 29 | 1.88 | 1.08E-03 |
| R0LN18 | Spectrin beta chain, brain 1 | SPTBN1 | 41.03 | 80 | 76 | 1.89 | 1.90E-11 |
| R0LLL3 | Early endosome antigen 1 | Anapl_08585 | 11.42 | 13 | 13 | 1.90 | 2.85E-02 |
| U3ICP9 | Uncharacterized protein | RHEB | 22.28 | 4 | 4 | 1.90 | 4.61E-02 |
| R0LTL8 | STE20-like serine/threonine-protein kinase | Anapl_11104 | 5.643 | 6 | 5 | 1.90 | 9.62E-03 |
| R0LQ61 | T-complex protein 1 subunit zeta | Anapl_01692 | 53.45 | 19 | 19 | 1.91 | 2.54E-03 |
| U3I7D6 | Cytoglobin | CYGB | 43.81 | 9 | 9 | 1.91 | 3.19E-02 |
| R0K6M4 | Peptidylprolyl isomerase | FKBP9 | 15.83 | 7 | 7 | 1.92 | 4.83E-02 |
| R0JQR6 | Tyrosine-protein kinase-like 7 | Anapl_13488 | 10.17 | 8 | 8 | 1.92 | 1.30E-02 |
| U3IR49 | Leucine rich repeat containing 40 | LRRC40 | 17.69 | 9 | 9 | 1.93 | 5.12E-03 |
| U3ID57 | Aminopeptidase | ANPEP | 25.71 | 18 | 18 | 1.93 | 9.76E-04 |
| U3IDM0 | Tyrosine--tRNA ligase | YARS | 46.37 | 22 | 22 | 1.93 | 9.70E-05 |
| U3I978 | Prolyl 3-hydroxylase 1 | P3H1 | 18.51 | 8 | 8 | 1.94 | 3.33E-03 |
| U3I0D6 | Clathrin heavy chain | CLTCL1 | 28.9 | 45 | 16 | 1.94 | 3.64E-03 |
| U3IRI9 | Collagen type V alpha 1 chain | COL5A1 | 5.595 | 7 | 7 | 1.95 | 4.16E-02 |
| U3ISN7 | Laminin subunit beta 1 | LAMB1 | 12.91 | 20 | 20 | 1.96 | 6.27E-04 |
| R0K4J6 | Ras suppressor protein 1 | Anapl_16457 | 26.47 | 6 | 6 | 1.97 | 3.96E-02 |
| R0LIV0 | 26S protease regulatory subunit 6A | PSMC3 | 42.78 | 13 | 13 | 1.97 | 9.51E-04 |
| U3INQ1 | Eukaryotic translation initiation factor 4H | EIF4H | 35.94 | 9 | 8 | 1.97 | 3.49E-03 |
| U3IRX7 | Autophagy related 7 | ATG7 | 4.476 | 3 | 3 | 1.98 | 2.20E-02 |
| U3IG63 | Vacuolar protein sorting 4 homolog B | VPS4B | 18.82 | 8 | 8 | 1.98 | 4.26E-02 |
| U3I047 | Isovaleryl-CoA dehydrogenase | IVD | 42.29 | 14 | 14 | 1.98 | 6.63E-05 |
| R0JJW5 | Collagen alpha-2(VI) chain | Anapl_09440 | 5.784 | 5 | 5 | 2.00 | 4.24E-03 |
| R0L6J4 | Lymphocyte-specific protein 1 | LSP1 | 15.27 | 4 | 4 | 2.02 | 8.96E-03 |
| R0JCP8 | Calcium-binding mitochondrial carrier protein SCaMC-1 | SLC25A24 | 11.75 | 5 | 5 | 2.05 | 4.97E-02 |
| U3IU67 | Calponin | CNN3 | 28.1 | 8 | 8 | 2.05 | 6.84E-04 |
| U3IHB9 | Inosine-5'-monophosphate dehydrogenase | IMPDH2 | 37.42 | 15 | 15 | 2.06 | 6.47E-03 |
| R0JA86 | Core histone macro-H2A.2 | H2AFY2 | 23.39 | 7 | 5 | 2.06 | 4.70E-02 |
| R0JDX1 | Coiled-coil domain containing 124 | CCDC124 | 23.98 | 4 | 4 | 2.06 | 3.22E-02 |
| U3I9Z9 | Talin 1 | TLN1 | 12.23 | 11 | 6 | 2.07 | 5.00E-02 |
| U3IID8 | Uridine monophosphate synthetase | UMPS | 35.9 | 13 | 13 | 2.07 | 1.44E-02 |
| U3J070 | Carboxypeptidase Q | CPQ | 23.03 | 8 | 8 | 2.07 | 3.23E-02 |
| U3IW58 | Collagen type I alpha 2 chain | COL1A2 | 32.94 | 29 | 29 | 2.07 | 6.56E-04 |
| U3IBP5 | Uncharacterized protein | LOC101804845 | 21.01 | 5 | 5 | 2.09 | 1.85E-02 |
| U3J3N7 | ENAH, actin regulator | ENAH | 7.864 | 7 | 7 | 2.09 | 6.89E-03 |
| R0JX52 | Calcium/calmodulin-dependent protein kinase type 1D | Camk1d | 17.31 | 5 | 5 | 2.09 | 8.78E-03 |
| U3IZH0 | Transgelin 2 | TAGLN2 | 56.35 | 7 | 6 | 2.10 | 4.00E-02 |
| R0L6A3 | Bifunctional 3'-phosphoadenosine 5'-phosphosulfate synthetase 1 | Anapl_04777 | 12.91 | 8 | 6 | 2.11 | 1.33E-02 |
| U3IF25 | Phosphatidylserine decarboxylase proenzyme, mitochondrial | PISD | 25.65 | 10 | 10 | 2.11 | 2.10E-02 |
| U3IRF4 | FMR1 autosomal homolog 1 | FXR1 | 7.046 | 4 | 3 | 2.14 | 4.43E-02 |
| R0L6Q1 | Delta-1-pyrroline-5-carboxylate synthetase | ALDH18A1 | 59.36 | 27 | 27 | 2.16 | 5.22E-04 |
| R0LVV1 | Basic leucine zipper and W2 domain-containing protein 2 | BZW2 | 18.73 | 8 | 6 | 2.18 | 5.88E-03 |
| U3J3H8 | RAB3 GTPase activating protein catalytic subunit 1 | RAB3GAP1 | 3.245 | 2 | 2 | 2.18 | 3.31E-02 |
| U3IT60 | Major vault protein | MVP | 22.55 | 6 | 6 | 2.18 | 3.65E-02 |
| U3J7D0 | Biphenyl hydrolase like | BPHL | 25.68 | 7 | 2 | 2.19 | 1.01E-02 |
| U3J6G2 | Tropomyosin 3 | TPM3 | 35.71 | 14 | 7 | 2.19 | 3.10E-02 |
| R0K419 | Heme oxygenase 1 | HMOX1 | 64.65 | 19 | 19 | 2.19 | 3.53E-04 |
| R0KFX1 | Heterogeneous nuclear ribonucleoprotein H3 | HNRNPH3 | 44.9 | 11 | 10 | 2.20 | 2.71E-02 |
| U3J2R5 | Lumican | LUM | 20.06 | 6 | 6 | 2.20 | 8.13E-03 |
| P00705 | Lysozyme C-1 | N/A | 21.09 | 3 | 3 | 2.21 | 3.82E-02 |
| U3IPJ8 | Transcription elongation regulator 1 | TCERG1 | 10.92 | 11 | 11 | 2.21 | 1.16E-02 |
| R0LBU5 | Dihydropyrimidinase like 2 | DPYSL2 | 39.64 | 20 | 14 | 2.21 | 2.56E-04 |
| R0LPZ6 | Thioredoxin domain containing 5 | TXNDC5 | 55.17 | 15 | 14 | 2.22 | 2.69E-03 |
| U3I4C0 | Tyrosine 3-monooxygenase/tryptophan 5-monooxygenase activation protein zeta | YWHAZ | 60.32 | 19 | 11 | 2.22 | 3.87E-02 |
| U3J9F2 | Septin 5 | SEPT5 | 21.65 | 7 | 7 | 2.23 | 9.70E-03 |
| U3IS46 | Laminin subunit alpha 5 | LAMA5 | 2.2 | 6 | 6 | 2.24 | 3.13E-03 |
| U3I589 | Tryptophanyl-tRNA synthetase | WARS | 20.68 | 8 | 8 | 2.25 | 1.59E-03 |
| U3INP6 | Calpain 5 | CAPN5 | 15.04 | 8 | 8 | 2.29 | 1.38E-02 |
| U3IYS1 | Carboxylic ester hydrolase | BCHE | 15.75 | 7 | 7 | 2.29 | 3.21E-03 |
| U3IU72 | 5'-nucleotidase ecto | NT5E | 19.76 | 9 | 9 | 2.30 | 9.91E-03 |
| U3IQT7 | UTP--glucose-1-phosphate uridylyltransferase | UGP2 | 42.44 | 20 | 20 | 2.31 | 1.61E-04 |
| U3I8S0 | Uncharacterized protein | N/A | 17.26 | 7 | 6 | 2.31 | 1.56E-02 |
| U3IX69 | Peptidyl-tRNA hydrolase 2 | PTRH2 | 75 | 5 | 5 | 2.31 | 4.09E-02 |
| U3IXM0 | Peptidylprolyl isomerase | FKBP4 | 39.81 | 15 | 15 | 2.31 | 6.92E-04 |
| R0JGG2 | 3-hydroxy-3-methylglutaryl coenzyme A synthase | HMGCS1 | 30.48 | 14 | 14 | 2.32 | 3.23E-02 |
| U3IEX4 | Adducin 1 | ADD1 | 13.45 | 9 | 9 | 2.34 | 4.95E-03 |
| R0LDL8 | Gamma-glutamyltransferase 5 | Anapl_06120 | 13.82 | 6 | 6 | 2.34 | 6.11E-03 |
| U3IJ97 | Fibrillin 3 | FBN3 | 2.078 | 6 | 5 | 2.35 | 1.24E-02 |
| U3IQE3 | Glycerol-3-phosphate dehydrogenase [NAD(+)] | LOC101798440 | 62.29 | 18 | 15 | 2.36 | 2.83E-04 |
| U3IB49 | Uncharacterized protein | LOC101795398 | 48.97 | 11 | 11 | 2.37 | 1.74E-05 |
| U3J007 | Peptidylprolyl isomerase | FKBP7 | 39.07 | 4 | 4 | 2.37 | 3.18E-02 |
| U3J8Y5 | Uncharacterized protein | LOC101803251 | 54.44 | 14 | 9 | 2.38 | 1.84E-03 |
| U3J091 | Kinectin 1 | KTN1 | 19.79 | 23 | 23 | 2.39 | 1.41E-03 |
| U3I4I9 | Actinin alpha 1 | ACTN1 | 52.72 | 27 | 22 | 2.41 | 7.15E-05 |
| U3IR26 | Vinculin | VCL | 49.72 | 46 | 46 | 2.41 | 3.26E-06 |
| U3ISE0 | Septin 11 | SEPT11 | 32.24 | 13 | 6 | 2.41 | 4.36E-03 |
| U3IM50 | Septin 9 | SEPT9 | 15.44 | 9 | 8 | 2.42 | 1.14E-03 |
| U3IRV2 | Dihydropyrimidinase like 3 | DPYSL3 | 21.75 | 11 | 7 | 2.43 | 1.55E-02 |
| R0L334 | Barrier-to-autointegration factor | BANF1 | 46.94 | 2 | 2 | 2.43 | 3.60E-02 |
| R0LCZ3 | Nidogen-1 | NID1 | 6.323 | 6 | 6 | 2.44 | 5.12E-03 |
| U3IP36 | Heat shock protein family A (Hsp70) member 9 | HSPA9 | 61.28 | 41 | 39 | 2.44 | 5.57E-08 |
| U3J7X3 | Peptidylprolyl isomerase | FKBP10 | 28.7 | 15 | 15 | 2.45 | 8.82E-06 |
| R0JKK6 | Collagen alpha-1(VI) chain | Anapl_09441 | 6.845 | 6 | 6 | 2.47 | 2.33E-03 |
| R0LPL6 | Asparagine synthetase (glutamine-hydrolyzing) | ASNS | 27.45 | 13 | 13 | 2.48 | 1.02E-05 |
| U3J730 | Ornithine aminotransferase | OAT | 53.9 | 22 | 22 | 2.51 | 7.02E-05 |
| R0J982 | Myosin-10 | MYO10 | 28.82 | 54 | 31 | 2.51 | 2.62E-08 |
| U3ISF4 | Succinyl-CoA:3-ketoacid-coenzyme A transferase | OXCT | 15.7 | 7 | 7 | 2.54 | 2.58E-03 |
| U3IC54 | Uncharacterized protein | N/A | 7.908 | 15 | 15 | 2.55 | 1.62E-04 |
| R0K048 | Gamma-tubulin complex component | Anapl_05331 | 3.016 | 2 | 2 | 2.55 | 3.42E-02 |
| R0M3G1 | Ectonucleotide pyrophosphatase/phosphodiesterase family member 3 | ENPP3 | 3.049 | 2 | 2 | 2.56 | 4.42E-02 |
| U3IY23 | Uncharacterized protein | N/A | 20.93 | 2 | 2 | 2.56 | 1.59E-02 |
| U3IR89 | Uncharacterized protein | CTSD | 49.75 | 23 | 23 | 2.59 | 1.92E-05 |
| U3I9J3 | Transcription factor BTF3 | BTF3L4 | 48.5 | 5 | 5 | 2.61 | 4.94E-02 |
| U3IFI6 | Cold inducible RNA binding protein | CIRBP | 31.4 | 8 | 7 | 2.64 | 3.92E-02 |
| R0JUU9 | Cytoskeleton-associated protein 4 | CKAP4 | 59.68 | 25 | 25 | 2.65 | 3.18E-07 |
| U3IR68 | Hexokinase 1 | HK1 | 12.47 | 11 | 8 | 2.68 | 2.36E-02 |
| U3IJ39 | AHNAK nucleoprotein | AHNAK | 19.83 | 26 | 26 | 2.76 | 4.85E-09 |
| U3I935 | Moesin | MSN | 42.04 | 27 | 19 | 2.77 | 5.40E-07 |
| U3J4Z3 | Nucleosome assembly protein 1 like 4 | NAP1L4 | 32.77 | 9 | 7 | 2.77 | 7.73E-03 |
| U3ICG4 | Osteoglycin | OGN | 14.57 | 4 | 4 | 2.77 | 1.50E-02 |
| U3J680 | Caldesmon 1 | CALD1 | 14.15 | 7 | 7 | 2.82 | 1.68E-04 |
| U3IKM2 | N-acylsphingosine amidohydrolase 1 | ASAH1 | 23.71 | 9 | 9 | 2.84 | 1.78E-03 |
| U3I5G2 | TP53 induced glycolysis regulatory phosphatase | TIGAR | 22.66 | 4 | 4 | 2.85 | 2.98E-02 |
| U3IQ16 | Solute carrier family 1 member 4 | SLC1A4 | 8.283 | 4 | 4 | 2.91 | 3.03E-02 |
| R0L1W0 | Lipoma-preferred partner-like protein | Anapl_09536 | 11 | 5 | 5 | 2.92 | 2.37E-02 |
| U3IFD3 | Myosin heavy chain 11 | MYH11 | 26.38 | 48 | 30 | 2.94 | 2.75E-12 |
| Q90240 | Carboxypeptidase D | CPD | 8.063 | 10 | 10 | 2.98 | 2.27E-04 |
| U3IUB7 | Lamin B2 | LMNB2 | 47.91 | 28 | 27 | 3.02 | 1.52E-07 |
| R0JQ38 | Tenascin | TNC | 8.287 | 14 | 14 | 3.03 | 1.60E-04 |
| U3J7P9 | Uncharacterized protein | LOC101803469 | 50.43 | 21 | 21 | 3.04 | 2.46E-06 |
| R0L0P0 | Creatine kinase B | CKB | 36.75 | 9 | 9 | 3.20 | 6.46E-05 |
| R0JFX3 | Elongation factor 1-alpha | EEF1A2 | 46.63 | 19 | 6 | 3.20 | 4.33E-03 |
| U3I0A3 | Keratin 7 | KRT7 | 46.54 | 22 | 14 | 3.22 | 3.53E-05 |
| U3IUR2 | Procollagen-lysine,2-oxoglutarate 5-dioxygenase 2 | PLOD2 | 23.25 | 13 | 13 | 3.27 | 2.01E-04 |
| R0K8D1 | Peroxidasin-like protein | PXDNL | 9.59 | 9 | 9 | 3.31 | 6.24E-03 |
| R0K6Y1 | SON protein | Anapl_04531 | 6.957 | 2 | 2 | 3.33 | 1.96E-02 |
| U3ISF1 | Phosphoinositide-3-kinase regulatory subunit 4 | PIK3R4 | 0.9545 | 1 | 1 | 3.43 | 1.74E-02 |
| U3IWZ8 | Phosphomannomutase | PMM2 | 41.03 | 9 | 9 | 3.44 | 1.76E-04 |
| U3I2P1 | Isocitrate dehydrogenase [NAD] subunit, mitochondrial | IDH3A | 28.88 | 8 | 8 | 3.48 | 6.67E-03 |
| U3J8Q5 | Uncharacterized protein | CALM1 | 58.11 | 7 | 7 | 3.49 | 1.20E-02 |
| U3I8W8 | Transforming growth factor beta induced | TGFBI | 11.68 | 8 | 8 | 3.50 | 9.26E-04 |
| U3IZT6 | Prolylcarboxypeptidase | PRCP | 12.33 | 5 | 5 | 3.51 | 2.70E-02 |
| R0LQ88 | Phosphoserine phosphatase | PSPH | 39.82 | 8 | 8 | 3.61 | 9.66E-05 |
| U3J364 | Galactosamine (N-acetyl)-6-sulfatase | GALNS | 9.581 | 5 | 4 | 3.81 | 3.08E-02 |
| U3IU76 | Uncharacterized protein | GARS | 57.71 | 9 | 9 | 3.86 | 6.79E-05 |
| U3I535 | Aldo-keto reductase family 1 member D1 | AKR1D1 | 42.64 | 11 | 11 | 3.91 | 4.98E-05 |
| U3I676 | Branched-chain-amino-acid aminotransferase | BCAT1 | 25.2 | 7 | 7 | 3.93 | 3.65E-03 |
| R0JFQ9 | Peroxiredoxin-6 | PRDX6 | 77.46 | 13 | 13 | 3.99 | 2.60E-03 |
| R0LLK8 | Decorin | DCN | 38.1 | 11 | 11 | 4.02 | 3.60E-04 |
| U3IY90 | Serpin family B member 10 | SERPINB10 | 16.67 | 7 | 7 | 4.04 | 1.76E-03 |
| U3I939 | Fructose-bisphosphate aldolase | ALDOA | 69.25 | 25 | 20 | 4.07 | 5.09E-05 |
| U3ILT8 | Collagen type XIV alpha 1 chain | COL14A1 | 18.44 | 30 | 29 | 4.08 | 3.68E-11 |
| R4HH66 | Desmin | DES | 44.7 | 13 | 8 | 4.16 | 3.12E-04 |
| U3IWD3 | Septin 2 | SEPT2 | 42.5 | 12 | 7 | 4.18 | 2.64E-03 |
| R0JVB0 | Dihydropyrimidinase-related protein 1 | Crmp1 | 13.18 | 8 | 5 | 4.37 | 1.18E-02 |
| U3I560 | Actin, alpha 2, smooth muscle, aorta | ACTA2 | 75.6 | 37 | 10 | 4.39 | 1.41E-04 |
| U3I027 | Glutamine--fructose-6-phosphate transaminase 1 | GFPT1 | 28.26 | 16 | 16 | 4.46 | 7.69E-06 |
| U3IES1 | Annexin OS=Anas platyrhynchos | ANXA2 | 35.59 | 11 | 11 | 4.49 | 1.37E-04 |
| U3IQ12 | Retinol binding protein 7 | RBP7 | 34.13 | 9 | 8 | 4.63 | 8.39E-05 |
| U3I0F9 | Pyruvate kinase | PKM | 49.91 | 25 | 25 | 4.74 | 3.80E-07 |
| R0JKM2 | Catechol O-methyltransferase | COMT | 64.12 | 13 | 13 | 4.79 | 3.53E-04 |
| U3J9B2 | Uncharacterized protein | N/A | 30.05 | 6 | 2 | 5.01 | 2.97E-02 |
| U3I6R1 | Annexin | ANXA6 | 49.85 | 36 | 33 | 5.09 | 1.27E-08 |
| U3ID88 | Collagen type VI alpha 3 chain | COL6A3 | 7.334 | 19 | 19 | 5.10 | 5.07E-06 |
| U3IRN3 | S100 calcium binding protein A12 | S100A12 | 36.97 | 4 | 4 | 5.27 | 5.60E-03 |
| U3ICJ6 | 3-hydroxybutyrate dehydrogenase 1 | BDH1 | 10.5 | 4 | 4 | 5.55 | 4.29E-02 |
| R0LNU6 | Periostin | POSTN | 27.64 | 16 | 16 | 5.74 | 6.99E-09 |
| U3IRY0 | Vimentin | VIM | 64.52 | 36 | 30 | 5.92 | 2.26E-09 |
| R0M4W3 | A-kinase anchor protein 12 | AKAP12 | 8.029 | 6 | 3 | 6.05 | 2.40E-02 |
| R0LM85 | Myosin light chain kinase | MYLK | 7.922 | 11 | 11 | 6.10 | 1.35E-05 |
| R0JPJ8 | Serpin H1 | SERPINH1 | 34.81 | 13 | 13 | 6.16 | 9.27E-05 |
| U3IU50 | G protein subunit alpha o1 | GNAO1 | 24.9 | 5 | 4 | 6.58 | 1.17E-02 |
| U3IU70 | Xanthine dehydrogenase | XDH | 2.06 | 2 | 2 | 6.59 | 3.75E-02 |
| R0LJZ9 | UMP-CMP kinase 2, mitochondrial | CMPK2 | 48.32 | 7 | 7 | 6.60 | 6.07E-04 |
| U3HYT7 | Cellular retinoic acid binding protein 1 | CRABP1 | 37.04 | 6 | 6 | 6.72 | 3.71E-05 |
| U3IDM3 | Perilipin | PLIN2 | 67.58 | 24 | 24 | 8.65 | 1.70E-13 |
| U3IS24 | Glucagon | GCG | 49.76 | 11 | 11 | 20.11 | 1.82E-08 |

^*^Fold change is expressed as the ratio of the riboflavin-deficient to control group. For diminished proteins, the fold change was transformed to the corresponding negative value.
